# Self-Medication as a Global Health Concern: Overview of Practices and Associated Factors—A Narrative Review

**DOI:** 10.3390/healthcare13151872

**Published:** 2025-07-31

**Authors:** Vedrana Aljinović-Vučić

**Affiliations:** 1 Medical Affairs Department, Jadran Galenski Laboratorij d.d., Svilno 20, 51000 Rijeka, Croatia; vedrana.aljinovic@gmail.com; 2 School of Medicine, Department of Basic and Clinical Pharmacology and Toxicology, University of Rijeka, Braće Branchetta 20, 51000 Rijeka, Croatia

**Keywords:** self-medication, prescription medication, over the counter medication, healthcare professionals, healthcare students, patients, COVID-19, antimicrobials

## Abstract

Self-medication is a subject of global importance. If practiced responsibly, self-medication represents a part of self-care or positive care of an individual or a community in promoting their own health. However, today’s practices of self-medication are often inappropriate and irresponsible, and as such appear all over the world. Inappropriate self-medication can be connected with possible serious health risks and consequences. Therefore, it represents a global health issue. It can even generate additional health problems, which will eventually become a burden to healthcare systems and can induce significant costs, which also raises socioeconomic concerns. Hence, self-medication attracts the attention of researchers and practitioners globally in efforts to clarify the current status and define feasible measures that should be implemented to address this issue. This narrative review aims to give an overview of the situation in the field of self-medication globally, including current practices and attitudes, as well as implications for actions needed to improve this problem. A PubMed/MEDLINE search was conducted for articles published in the period from 1995 up to March 2025 using keywords “self-medication” or “selfmedication” alone or in combinations with terms related to specific subthemes related to self-medication, such as COVID-19, antimicrobials, healthcare professionals, and storing habits of medicines at home. Studies were included if self-medication was their main focus. Publications that only mentioned self-medication in different contexts, but not as their main focus, were excluded. Considering the outcomes of research on self-medication in various contexts, increasing awareness of responsible self-medication through education and informing, together with surveillance of particular medicines and populations, could lead to more appropriate and beneficial self-medication in the future.

## 1. Introduction

Self-medication is the selection and use of medicines to treat self-recognized illnesses and symptoms. The World Health Organization (WHO) defines self-medication as a practice that “…involves the use of medicinal products by the consumer to treat self-diagnosed disorders or symptoms, or the intermittent or continued use of a prescribed drug for chronic or recurrent disease or symptoms” [1] (p. 9). It represents a part of self-care, a term more broadly defined by WHO as the capacity of the population to stimulate health, prevent and control disease, and to deal with diseases and disability with or without the support of a healthcare provider [2]. The “consumer” in this aspect represents a layperson who is not educated in diagnosing diseases or medical conditions, nor in the use of medicinal products (medicines, drugs, and medications). Self-medication in these cases involves various practices, such as individuals recognizing symptoms and subsequently choosing the treatment that they autonomously deem appropriate, or situations where an individual’s condition has previously been diagnosed by a physician, and the individual later decides to use previously prescribed medicines all by themselves. Since self-medication practices are common in socioeconomically different societies, the subject has become a topic of growing interest in the global healthcare community.

According to the World Medical Association (WMA), self-medication is perceived as responsible when individuals treat their ailments and conditions with medicines that are approved, available without prescription, and which are effective and safe when used as intended [3]. Self-medication can be beneficial to both the patients and the healthcare system in situations where patients self-medicate themselves in case of some minor ailments instead of putting additional burden on already overwhelmed health practitioners [4,5]. Clearly, it is crucial that patients recognize their health condition and are able to choose the right medicine and administer it in a proper and responsible way to treat the condition. That would represent an ideal case of self-medication. On the other hand, irresponsible self-medication can pose a risk, potentially even a serious threat to one’s health in various ways [4,5,6,7,8]. Individuals who do not recognize the ailment can misinterpret and misdiagnose their condition, which can lead to taking the wrong medicine, one that will not treat the real disease, and the disease can progress while a person is exposed to potential risks associated with the prolonged use of the wrong medicine [4,5,6]. Also, by self-medicating themselves, it sometimes happens that patients mask early symptoms of the disease, therefore delay seeking proper help from healthcare professionals (HCPs), which can be especially dangerous when a person has a serious disease [5]. Furthermore, if individuals are not aware of possible risks or adverse drug reactions (ADRs) connected with medicine use, which also refers to non-prescription medicines, or are not aware of proper medicine usage and take the wrong dose of a certain medicine or take it concomitantly with their chronic therapies, this can lead to possible health problems and result in organ injuries [5,6,9,10,11,12,13]. Underdosing leads to falling short of the needed efficacy, and overdosing leads to potential toxicity, which can cause more health problems [5,13,14], including those serious enough to require hospitalization [15]. In the most severe cases, inappropriate self-medication can also cause death, even when using common over-the-counter (OTC) medicines that are used for headaches, such as paracetamol [14,16]. The question of the potential consequences of self-medication is a pressing issue.

There are multiple possible motives for a person to start self-medication, such as an overwhelmed healthcare system where providing care can sometimes be hindered, the number of patients, overwhelming working conditions, or a lack of time needed for a physician’s check-up [17,18,19,20]. Another plausible reason to get engaged in self-medication practice is the enhanced availability of medicines because of the switch of many molecules from prescription to over-the-counter (OTC) status. As a result of switching from prescription drugs to OTC status, medicines that were more strictly regulated previously became more accessible to patients [21,22]. The availability of medicines through online pharmacies additionally aided access to medicines, with benefits such as enabling consultation with HCPs and receiving medicines from the comfort of patients’ homes, offering greater privacy for patients who feel a lack of it when discussing certain health issues in community pharmacies, and lower prices of medicinal products in some countries [23]. Nevertheless, one of the issues that raises big concern is that, according to some resources, many websites that provide prescription-only medicines operate illegally [23]. Also, online pharmacies could avoid the criteria that regulators impose on standard pharmacies. Hence, some of them could engage in dispensing prescription-only medicines without the prescription itself, which can pose a serious threat to patients’ health in a variety of ways that relate to unnecessary or inappropriate use of medicines [24]. Additionally, there is a possibility that some illegally operating online pharmacies would even dispense medicines that are fake, expired, or not stored properly, which is a serious issue that poses health and legal concerns [24]. Inappropriate self-medication today constitutes a public health concern and socioeconomic challenge. It is a serious issue that aggravates health-related problems and possibly imposes additional costs on the healthcare system. This review tackles relevant current global health issues. Its aim is to outline and evaluate the relevant research carried out globally regarding self-medication and to give insight into the situation and elucidate research in areas such as practices of self-medication, attitudes toward it, and knowledge on the meaning of responsible self-medication or the lack of it. By analyzing international literature, this review gives an overview of global practices, motives, and risks for self-medication. Inclusion of various populations and contexts, such as the COVID-19 pandemic and changes in health-seeking actions in the post-pandemic era, provides richness to the analysis. The review seeks to explore the need for practical interventions in the field of self-medication and to formulate useful and constructive recommendations for the future, based on reviewed literature, which could be integrated into efforts aimed at risk prevention to subsequently improve the situation, considering irresponsible self-medication, minimize its consequences, and ensure appropriate and responsible self-medication.

The situation in the field of self-medication is examined and discussed by analyzing studies regarding self-medication with a special review of main themes: self-medication in the general population of both adults and children, self-medication and the COVID-19 pandemic, self-medication with antimicrobial drugs, self-medication in healthcare professionals and students of health studies, and the association between self-medication and keeping medicines at home. In the overview, studies are grouped according to their main theme. In cases when more themes overlap in one study, studies are categorized into groups according to their main focus. This study adopted a narrative review approach to ensure a broad understanding of the subject. As such, it provides an interpretation of the situation over the last three decades without the restrictions of strict, rigid inclusion/exclusion criteria or standardized methodological quality assessment typically found in systematic reviews. The review is based on an analysis of peer-reviewed English-language studies on PubMed/MEDLINE published in the period from 1995 up to March 2025. The non-systematic search (PRISMA guidelines not followed) was performed using a combination of keywords associated with self-medication. Search terms involved “self-medication” or “selfmedication” alone or in combinations with terms “COVID-19”, “antimicrobials”, “antibiotics”, “health care professionals”, “health care students”, “home medicines cabinets”, “home pharmacies”, and “home medical inventories”. Studies were included if they were original research articles published in peer-reviewed journals in the above specified period and were conducted with the scope of examining practices, attitudes, and/or opinions of self-medication per se and in various contexts, such as self-medication in COVID-19, self-medication with antimicrobials, self-medication in current and future healthcare professionals, and self-medication and the contents of home medicine cabinets/home pharmacies. Studies were excluded if they were published before 1995; only mentioned the term “self-medication/selfmedication” in diverse contexts, but it was not their main focus; or were not original research articles. Additional references were identified through manual searches of the reference sections of retrieved articles. All included studies, along with an overview of study features and findings, are shown in Supplementary Materials. Due to the narrative nature of this review, the quality of the included evidence was not systematically estimated, and formal risk-of-bias assessment and formal statistical analysis were not performed. Whilst there was no following of a formal systematic review protocol, dedication to transparent choice and assessment ensured a structured and robust narrative review.

## 2. Self-Medication in General Population

Self-medication is spread rather globally. Many articles describe the issues connected with irresponsible self-medication and the need for intervention programs to improve the situation. As facilitators of self-medication, studies identified the impact of healthcare systems on self-medication practices, such as visits to doctors, long waiting lists to get medical check-ups, and an inability to afford medical evaluation and/or treatment [17]. Besides reasons connected to health systems, the impact on the decision to start self-medication is also associated with taking advice from other people, such as friends and family who are not HCPs, receiving information about the treatment online, and the ability to self-medicate the ailment/condition [25]. Persons with chronic health conditions, as well as persons who believe they can choose the right treatment, are also inclined to administer drugs to themselves without a consultation with health professionals [25,26,27]. People who are well educated, including those who pursue education in healthcare, were shown in certain studies to be more inclined to ask for advice from physicians and pharmacists [26,28]. Analgesics, antipyretics, and non-steroidal anti-inflammatory drugs (NSAIDs) were the medicines that were most frequently self-medicated in several studies [29,30,31,32,33,34]. In the context of opinions and attitudes on self-medication, the possibility of medicines causing adverse events is considered a limitation to self-medication, as well as the possibility of particular medicines causing a habit [26]. However, what is rather worrisome is that in certain cases, even when knowing that self-medication can pose a defined risk, people still practice it, even with children [35,36]. Yeamans et al. found that the prevalence of self-medication in the European Union (EU) was 34.3% [17]. However, the prevalence was different between EU member countries, as was self-medication inequality between men and women. The study identified certain parameters associated with higher levels of self-medication, such as the age of individuals between 25 and 44, higher levels of education, immigrants born in other EU countries, and inhabitants of cities. The facilitators of self-medication were also chronic health conditions, visits to doctors, long waiting lists to get a medical check-up, and the inability to afford medical evaluation and/or treatment. Kloda et al. conducted a study to assess the opinion of outpatient healthcare physicians about the reasons patients start self-medication [25]. There were three main reasons the study identified for starting self-medication: taking advice from other people (friends and family who are not HCPs) (59.1%), finding information about the treatment online (52.9%), and the ability to self-medicate the ailment/condition (51.6%). Results showed that antibiotic treatment was self-medicated in an alarmingly high 72.1% of adult patients and in 39.8% of children. Additionally, children’s custodians were more inclined to visit physicians after the first symptoms (42.2%) than adult patients (22.1%) [25]. In a study by Gebert et al. on opinions about self-medication in younger and well-educated people, it was found that 59.3% of participants used self-medication in the previous 12 months on a regular basis [26]. The main symptom of self-medication was headache in 86.2% of surveyors, and complaints/symptoms were mild (94.7%). The possibility of self-medication to cause adverse drug reactions was perceived as the main identified risk in 94.2%, and the possibility of developing a habit was perceived as a risk in 58.7% of them. Regarding the sources of information on proper medication, 93.7% of surveyors listed pharmacists, and 89.3% of them listed physicians’ recommendations as “influencing factors”. More than half of the participants (61.3%) believed that they themselves could choose the right treatment. Barrenberg et al. calculated seven-day OTC drug prevalence and found that it was higher in women than in men (47.16% vs. 33.17%) [27]. Found predictors of OTC drug use included female gender, age above 60 years, lower health status, the use of prescription medicines, and multiple chronic conditions. The study also examined the levels of OTC drugs surveyors used during the previous seven days and found that levels of use of these drugs were lower than the level of self-medication, which was attributed to the possibility that some of the OTC drugs found were also recommended by physicians instead of being self-medicated by surveyors. Klemenc-Ketis et al. conducted research on self-medication in healthcare and non-healthcare university students during the previous year [28]. The majority of students (92.3%) from both groups, healthcare and non-healthcare studies, reported some kind of self-medication over the surveyed period. Healthcare students’ opinion was that self-medication without improvement in symptoms should last no more than a week. They obtained medicines for self-medication at pharmacies. A more important reason for self-medication was a physician’s advice in a previous similar situation. Patients would seek recommendations on different methods of treatment from a physician or pharmacist, and they perceived self-medication as not very safe. On the other hand, non-healthcare students acquired medicines from healers and friends. In a study by Kiroglu et al., the authors evaluated the practices of self-medication with conventional and herbal medicines among ear, nose, and throat (ENT) outpatients on their first visit to the otorhinolaryngology department [29]. Self-medication with conventional drugs before visiting the hospital was reported in 44.8% of respondents, with analgesics (31.7%) and antibiotics (21.9%) being the most common self-medicated drugs. It was reported that almost half of the surveyors (49.2%) used at least one herbal medicine, but 22.9% of respondents were not aware of an interaction of herbal drugs with other medicines.

In addition to the studies that included the adult population, a certain number of studies were performed on the self-medication practices of parents or custodians of their children. In the previously mentioned study by Kloda et al., besides adult patients, it also considered children [25]. Results showed that although children’s carers were more likely to visit physicians immediately with symptoms (42.2% of cases) than adult patients (22.1%), the decision to self-medicate antibiotics to children was made in 39.8% of cases. Research on practices of parents or custodians of self-medication of children by Tarciuc et al. found a significant relation between the beliefs of carers and their willingness to self-medicate children without previous medical consultation [36]. Results showed that the percentage of parents who self-medicated their children was 70%. A significant relation was reported between the number of illnesses experienced by children in the previous six months and the inclination of their parents/custodians to self-medicate them. As already mentioned, it was observed that when parents showed knowledge of self-medication risks, it did not stop them from self-medicating their children [36]. In a study by Martin-Perez et al., the results showed that during the two weeks before the survey, 8.2% of children received OTC medicines by their parents, among which the most commonly used were analgesics (30.3%), medicines for cold (25.5%), and antipyretics (22.8%) [30]. Regarding parameters that were predictors of self-medication, a study found that they included children in older age groups (10–14 years old), no chronic illness, children with limitations of normal activity, parents of middle or higher social status, and the completion of secondary education or higher. The study performed by Garofalo et al. found that self-medication of children without a physician prescription was practiced at least once in 69.2% of respondents [31]. Parameters associated with a higher probability of self-medication were female gender, younger population, and health problems in the preceding year. Those associated with a lower probability of self-medication were middle or lower levels of school education. More frequent use of self-medicated drugs in the previous year was disclosed for NSAIDs, and two-thirds of respondents disclosed inappropriate medication in the last year at least once. The overall results showed that the frequency of oral self-medication was rather high and inappropriate, which poses a reason for concern [31]. The use of self-medicated drugs in children was shown to be more frequent for analgesics/NSAIDs, and the majority of respondents reported inappropriate medication use in the last year at least once [30,31]. In research conducted by Du et al. on the prevalence and correlations of self-medication in outpatient children in the week before participation in the study, findings revealed that 25.2% of participants self-medicated (17% used OTC drugs and 9.9% used other sources of drugs) [37]. Regarding total medicine use, 38.5% was self-medication, which included all medication classes. Drugs that were most frequently self-medicated were those that act on the respiratory system (32.1%), gastrointestinal tract (GIT) and metabolism (21.6%), skin (14.2%), and nervous system (11.3%). Determinants that were closely associated with self-medication were older adolescent ages (14–17 years), poor health status of children, no immigration background, higher household income, and having mothers with higher educational levels [37]. Jenssen et al. conducted a study to determine the connection between maternal self-medication and self-medication of OTC medication among school children. Results showed that maternal use of OTC analgesics was significantly associated with OTC analgesic self-medication in children, especially paracetamol, perhaps even more than the child’s pain, while maternal health did not have a significant influence [38]. Relevant studies related to self-medication in the population are shown in Table 1.

## 3. Self-Medication During COVID-19 Pandemic

During the COVID-19 pandemic, common circumstances substantially changed. The lockdown was imposed, and access to health services was restricted. People were advised to stay at home to avoid the risk of getting an infection and to avoid putting an additional burden on exhausted healthcare providers. However, at the same time, people got sick from diseases other than COVID-19, and they needed help. In a number of cases, they needed pharmacological treatment. In many cases, limited access to HCPs stopped patients from seeking help directly from HCPs, and in some cases, patients themselves feared getting the disease from healthcare staff who were exposed to COVID-19 in their everyday contact with patients [19]. Receiving medical advice over the telephone became common, and many sought medical advice on the internet, which became loaded with news on medicines that were investigated for their potential effectiveness in COVID-19. Besides that, the inaccessibility of direct contact and check-ups with healthcare professionals led to an expansion in seeking health information online. All of this resulted in an increased rate of self-medication during the COVID-19 pandemic [19,43]. Some people who feared prolonged HCP inaccessibility saw stocking of medicines as a possible solution, and they bought significant amounts of particular OTC drugs, such as analgesics/antipyretics, as well as prescription drugs such as hydroxychlorochine and antibiotics, which they used for the prevention and treatment of COVID-19 [44,45]. Staying at home, as recommended, was, in a way, a good solution for patients to avoid being exposed to the potential risk of acquiring COVID-19 when visiting hospitals, outpatient clinics, or pharmacies. Additionally, many patients’ choice to self-medicate instead of seeking HCPs’ help for minor ailments [46] represented a substantial relief on the healthcare system, and it gave HCPs much-needed time to concentrate on the growing number of COVID-19 patients in that moment. However, in some patients, this practice resulted in a habit to self-medicate and stock drugs at home over time [44,45,47]. Research by Makowska et al. on self-medication-associated behavior during COVID-19 showed that during the three months of lockdown, 45.6% of respondents reported at least one behavior that could be regarded as inappropriate self-medication, including 16.6% who took medication as a safety measure, and 16.8% who took prescription medicine without consultation. The reason for concern was that some of these respondents had never exhibited such behavior prior to lockdown, and some of them engaged in this behavior for the first time during the lockdown [43]. In some cases, during the COVID-19 pandemic, research showed that approximately one in ten respondents in especially sensitive groups of people, such as users of drugs, reported stockpiling drugs, and their behavior was positively associated with reporting being heavily impacted by COVID-19 [45]. Mothers also reported giving OTC medicines to their children with the purpose of the prevention and treatment of COVID-19, and the increased probability of self-medication of OTC medicines was additionally connected to maternal self-medication [47]. Moreover, Karami et al. showed that during the pandemic period, the prevalence of intentional exposure to nonprescription analgesic/antipyretic medicines increased among females 6–17 years old [48]. A study by Mejia et al. found a significantly high prevalence of self-medication reported among respondents, which also included the use of medicines such as chloroquine, hydroxychlorochine, and antibiotics [49]. Studies showed that during the COVID-19 pandemic, OTC and prescription medicines were self-medicated for the prevention and treatment of COVID-19 [46,49,50]. This included self-medication with antibiotics [19,49,50,51,52,53,54,55,56,57], which was also on the rise, mostly in conditions where they are not effective, such as flu-like symptoms related to COVID-19 [52,54], which showed the influence of the COVID-19 pandemic on the increase in irresponsible self-medication, which further expanded the development of antimicrobial resistance.

Another issue that arose during COVID-19 in terms of self-medication was the increase in news on the potentially beneficial effects of particular medicines in treating the disease. Many people wanted to get ahold of these medicines (e.g., chloroquine, hydroxychloroquine, and azithromycin), which not only posed a risk of irresponsible self-medication with these medicines due to a fear of the consequences of disease but also reportedly caused stock-out of some of them, impairing the normal functioning of supply chains, which, as a consequence, impaired usual treatment patterns in specific therapeutic areas, such as rheumatology [58]. However, this aspect of the COVID-19 pandemic’s effects is not within the scope of this review. Relevant studies related to self-medication during the COVID-19 pandemic are shown in Table 2.

## 4. Self-Medication with Antimicrobials

Self-medication with antimicrobial medicines is a topic of particular concern in the context of self-medication. The overuse and misuse of antimicrobial drugs in humans, but also in animals and plants, are the main facilitators of pathogen resistance development or antimicrobial resistance (AMR) [59]. The alarming consequences of drug resistance development include the ineffectiveness of antimicrobial drugs, which makes treating infections harder; the spread of infectious diseases easier; and makes other medical procedures much riskier, such as surgeries, organ transplantations, and chemotherapy cancer treatments [59]. All of this can result in severe disease, disability, or death [59]. Regardless of attempts by the scientific community and pharmaceutical industry, the pipeline of new antibiotics is not abundant enough to cope with the rapid increase in microbial resistance. Estimates indicate that in 2019, bacterial AMR was an explicit reason for 1.27 million deaths globally and contributed to 4.95 million deaths [60]. In addition, AMR creates significant costs for both healthcare systems and countries’ economies.

The development of AMR is one of the risks that normally occur when patients practice irresponsible self-medication with antibiotics and other antimicrobial medicines. Accordingly, there are studies performed specifically on the subject of self-medication with antimicrobials or antibiotics. Literature research shows a concerning level of self-medication with antimicrobials, clarifying the high level of insufficiency of knowledge on both self-medication and proper antibiotic use, as well as the growing need for more education and public awareness campaigns. Reported levels vary between studies, and in some studies included in this review, researchers found that the rates of respondents who engaged in self-medication reached approximately 30–40% [41,51,52,61,62,63,64,65,66]. However, in some cases, levels of self-medication with antibiotics reached around two-thirds of the population examined and even more [25,67,68,69]. Vaananen et al. reported some worrisome findings, such as that 41% participants of the study bought their antibiotics without a prescription, as well as that the main reason for antibiotic use was a common cold in 45% of respondents, which was followed by a sore throat in 17% of respondents [62]. As mentioned previously, in a study by Arboleda et al., participants reported that they used antibiotics without a medical prescription for the treatment of flu-like symptoms associated with COVID-19 [52]. A survey on the use of antimicrobial drugs in 19 European countries conducted by Grigoryan et al. showed that the most common reasons for self-medication with antimicrobial drugs were throat symptoms and that the sources of commonly used antimicrobial drugs included pharmacies and leftover medicines from previous infections [70]. Another study conducted in 11 European countries to assess, among others, attitudes concerning the suitability of self-medication with antibiotics, situational use of antibiotics, and the awareness of AMR showed that awareness of AMR was the lowest in countries that had a higher prevalence of AMR [71]. Higher antimicrobial self-medication levels could be attributed to a lack of knowledge about AMR. It was reported in a study by Napoletano et al. that the definition of antibiotic resistance was known to only 9.8% respondents, while appropriateness on when to use antibiotics was known to 21.2% [72]. Approximately one-third of respondents reported that self-medicated antibiotics and factors associated with a higher use of self-medication were lower self-rated health status, not using a physician as the source of information on antibiotics, and visits to a physician within the last year. Of those who never self-medicated antibiotics, 22.7% were willing to take antibiotics without a physician’s prescription [72]. Self-medication with antibiotics and keeping antibiotics at home is also common among university students [63,67]. The level of self-medication with antibiotics, together with the lack of knowledge on their proper use, is a reason for serious concern. In a study by Zhu et al., almost half of students (47.9%) reported a life-time history of self-medication with antibiotics [67]. Healthcare students who independently used antibiotics believed that antibiotics were suitable for viral infections (43.5%), they used it more than once in the previous year (65.9%), and 13.3% experienced adverse drug events while using antibiotics without a prescription [67]. Wang et al. reported that 63.1% of surveyed students kept antibiotics at home. In 27.8% of cases, these antibiotics were leftovers from previous prescriptions, and two-thirds were bought without a prescription. Students who kept antibiotics at home were 5 times more likely to engage in self-medication with them and 2.6 times more likely to use antibiotics as prophylaxis [63]. Similar research on the use of non-prescription antibiotics in older populations showed low levels of knowledge on the proper use of antibiotics, and one-third of older adult respondents did not know that antibiotics only treat bacterial infections [73].

These findings in different population groups clarify the high level of insufficiency of knowledge on both self-medication and proper antibiotic use, as well as the growing need for more education and public awareness campaigns. Relevant studies related to self-medication with antimicrobials (antibiotics) are shown in Table 3.

## 5. Self-Medication in Healthcare Professionals

Self-medication in special groups of populations, such as healthcare professionals and university students of future health professions, is a peculiar subject. Since healthcare providers should be the primary resource of counseling on appropriate and responsible use of medicines, and students of healthcare studies are the ones who will assume that role in the future, it is interesting to examine the level of self-medication in these groups. Self-treatment by healthcare professionals is an issue that draws special attention, even in professional associations. In that aspect, the guidelines of The United Kingdom (UK) General Medical Council (GMC) recommend that, to the extent possible, physicians should not treat themselves nor the members of their families or others with whom they have close personal connections [77]. It is particularly emphasized that they should not prescribe controlled substances to their relatives or themselves [77]. The Code of Medical Ethics of the American Medical Association (AMA) also discourages physicians from treating themselves or close family members, stating that the physician’s professional objectivity in such cases may be compromised [78]. Therefore, the topic of self-medication in healthcare professionals and in students of health studies who will become HCPs someday is of particular interest.

Some studies found that having a member of the household who is in a healthcare/medical profession was associated with a higher level of knowledge on the appropriate use of medicines [72]. An analysis of selected studies on self-medication that were conducted among HCPs or students of healthcare studies who are exposed to learning the proper use of medicines in their education was performed. Research results show that healthcare professionals are reluctant to seek help in areas of mental health because of the fear of implications for their career, stigma, and professional standing [79,80,81,82,83], and because of that, they engage in self-medication with medicines that treat mental conditions [79]. Asut et al. investigated psychiatrist and psychiatry residents’ engagement in self-medication in Turkey and found that 83% of participants were engaged in self-medication, and although 80.9% percent of them reported knowledge of self-medication, many of them lacked awareness of legal and ethical guidelines. As major reasons for practicing self-medication, the participants reported that their ailment was minor, they had a previous positive experience with self-medication, and there was a lack of time to seek help from another healthcare professional. They also tried to avoid the stigma that is often associated with psychiatry and psychiatry residency [79]. In addition, it is common that physicians or pharmacists engage in self-medication of other medicines, such as antibiotics and dermatology medicines [54,84,85,86,87].

In this regard, students of various health science faculties who obtain knowledge in pharmacology during their studies constitute a specific research group. Self-medication is also observed in university students of healthcare studies, such as medicine, pharmacy, and nursing [28,88,89,90,91,92,93,94,95,96]. In the future, many of these students will probably have a role in prescribing drugs, giving advice on the appropriate use of drugs, and shaping drug policies, and it is of particular interest to get to know their self-medication practices and knowledge on the proper use of medicines, as well as their beliefs and attitudes toward self-medication.

It was mentioned earlier that, although healthcare students practiced self-medication, they also showed knowledge of possible risks of that practice and asked for advice in proper treatment from physicians or pharmacists [28]. The most common symptom for self-medication among healthcare students was headache [90,91,93,96], and the most frequently self-medicated medicines were paracetamol/analgesics/NSAIDs [88,90,91,92,93,96]. However, the practice of self-medication with antibiotics was also common among healthcare students [89,90,94]. A study by Benameur et al. found that 79.9% of medical students were aware that self-medication with antibiotics is not safe and is inappropriate [89]. Nevertheless, the level of self-medication with antibiotics remained high. Concerning the attitudes toward self-medication and engaging in that practice, familiarity with the health issue or perceiving the symptoms as minor or simple were among the reasons that healthcare students engaged in self-medication [88,90,93], as well as perceptions of their knowledge on medication [91,93]. Relevant studies related to self-medication in healthcare professionals or students of healthcare studies are shown in Table 4.

Additionally, though self-medication with psychotropic medicines is not in the narrow focus of this review, it is worth mentioning that not only HCPs but also other groups in the population show an inclination to practice it. This can be dangerous, especially in vulnerable populations, such as adolescents, the elderly, or mentally unstable patients. Self-medication with medicines such as sedatives, anxiolytics, and antidepressants raises concern because, besides the risk of their toxicity when taking a higher dose and their potential to develop a habit, these medicines can also cause serious long-term issues and can be associated with suicidal ideas and risks. People who try to self-medicate with these types of drugs sometimes do it to hide the fact that they need this kind of medicine because of potential stigma in society. However, since inappropriate self-medication with prescription psychotropic medicines in students and adolescents can cause the above-mentioned problems, it raises serious concern, for it has also been shown that it may promote suicidal ideation and risk [98]. Nonetheless, even self-medication with any prescription drug could pose a risk of suicidal ideas and attempts [99]. This is not an issue exclusively for the younger generations but also for older population, where it has been found that the inappropriate use of prescription benzodiazepines and opioids was associated with suicidal ideations, and HCPs should be aware of these risks [100].

## 6. Self-Medication and Medications Kept in Home Pharmacies/Medicine Cabinets/Drug Inventories

An issue very closely connected to self-medication is what can be found in terms of stocking medicines at home and in investigating the contents of home pharmacies/drug inventories/medical cabinets over the years. Besides a higher probability of engagement in self-medication for people who keep medicines (antibiotics) at home, other studies showed that among medicines usually kept at home, the largest proportion were nonopioid analgesics and non-steroidal inflammatory drugs (NSAIDs) [63,90,95,101,102,103,104], and self-medication was a common practice with both prescription and OTC medicines [88,95,101]. People who keep medicine inventories also keep antibiotic drugs [63,95,103,104], as well as drugs that are expired or drugs with unknown purpose, to give to household members [95,105,106]. Keeping medicines at home seems to be a common practice, and people who usually self-medicate have larger inventories of medicines stored at home, including antibiotics [101,102,103,104,106]. Relevant studies related to self-medication and storing medicines at home are shown in Table 5.

## 7. Discussion

Present overview of the research shows that self-medication remains a growing healthcare issue globally. Studies analyzed in this review show a worrisome level of self-medication among the general population, as well as among healthcare professionals. Although rates of reported self-medication vary among various studies and different countries and conditions, overall, they are rather high for both adults and children. Circumstances, including catastrophic events such as the COVID-19 pandemic, stimulated people to practice self-medication even more, and, in addition, to stockpile drugs at home. Nevertheless, keeping hoards of drugs at home seems to be a behavior that facilitates practicing self-medication independently of the pandemic. Drugs often used for self-medication are various and include alarming examples, such as antibiotics and psychotropic drugs. The former were shown to be self-medicated by many, even though antimicrobial resistance is a globally recognized phenomenon, while self-medication of the latter is particularly worrisome among the more sensitive, possibly suicidal population. As expected, analgesics/antipyretics were often found, which should also be regarded in the light of possible serious risks associated with their use. This shows that self-medication is a complex issue that depends on many various intertwined variables. For self-medication to be appropriate and responsible and to represent a valuable part of self-care, there should undoubtedly exist a certain level of knowledge that the user possesses on medicines and their possible risks. Regarding necessary knowledge, when it comes to the people who certainly have it, such as healthcare professionals and healthcare students, they, as expected, do show a higher overall level of knowledge of the appropriate usage of medicines. However, in many studies, it was found that despite their good education in the field of medication management and their positive attitudes toward appropriate and responsible self-medication, they still do engage in it, even with antibiotics. In addition to what was previously mentioned regarding keeping medicines at home, an inspection of home medical cabinets showed that medicines kept at home were both used and unused. These medicines were kept for future use based on the patients’ own estimation and previous experience when a medicine was previously prescribed by a physician. Keeping expired medicines and those for which the purpose is unknown, as well as keeping them within the reach of children, are all signs of irresponsible practices in this aspect. Possible consequences of irresponsible self-medication are many, and there are various possibilities that could help to tackle the problems that could arise from it. Insights into the selected research conducted globally imply the growing necessity for immediate actions directed toward increasing the awareness of responsible self-medication.

The coordinated efforts of different health professions are paramount. Regarding the public, there is an alarmingly high level of all kinds of medicines used without HCPs’ advice, both prescription and OTC. Increasing the consciousness of the public about possible self-medication risks is urgently needed, and it could be strengthened through the development of customized educational programs and activities, such as public health campaigns, as well as by means of health institutions and primary caregivers employing a more proactive approach. This refers specifically to family physicians and community pharmacists as supervisors of medication usage and educators of patients. In order to better control self-medication and prevent its inappropriate habits, comprehensive practical interventions should be developed and directed to minimize identified risks. Understanding the reasons and behaviors related to self-medication is necessary to develop scientifically supported methods for reaching these goals. Developed interventions should include enhanced access to reliable sources of information, such as more accessible pharmacists, who can counsel patients and assist with proper self-medication, and educational programs, which would provide information on appropriate medication use and potential side effects, as well as guidance on when patients should visit a physician. The extent of the actions implemented can vary from simple actions, such as information leaflets, to more complex ones, such as educational workshops for the public. Additionally, in strengthening positive self-medication practices, digital health tools could be useful in a variety of ways: trustworthy websites with content created by HCPs can be a reliable source of useful information for patients in need of advice on responsible self-medication. Furthermore, easily accessible digital applications should be developed with HCPs’ inputs to specifically assist diverse self-medication situations, various patient groups, and different medications; ensure providing appropriate information; or to act as a reminder for proper drug use. However, in order to fully integrate digital health tools in the self-management of diseases, both acute and chronic, further research is needed to evaluate the effectiveness of implemented interventions and to generate reliable evidence to ensure optimal patient outcomes.

To improve the status of self-medication in the future, further specific research on the subject is warranted to acquire more detailed knowledge on its reach and its impact in different aspects and distinct population groups.

## 8. Recommendations

There is an overwhelming need to develop and implement educational programs aimed at educating both HCPs and the public. Considering HCPs’ education, although they obtain most of the information needed from their educational curricula, specific, custom-tailored programs could be a useful addition. Particularly useful could be the possibility to discover potential self-medication when prescribing or dispensing medicines, and, in the case of relevant self-medication being disclosed, HCPs could control better the use of OTC medicines, to the extent possible, to prevent unnecessary and inappropriate self-medication by patients [108]. A summary of practical recommendations from reviewed studies is shown in Table 6.

## 9. Limitations

The limitations of this review are primarily due to its narrative structure since, in non-systematic review, selection bias can be present in the inclusion of articles, and key studies might be missed. The selected studies had diverse methodologies, sample size, and a diverse number of variously composed questionnaires, as well as various forms of evaluation by authors, which limits the comparability of findings. Additionally, the quality of the included evidence was not systematically estimated, but the vast majority of the retrieved and included articles were cross-sectional studies, which are generally regarded as providing a lower level of evidence. Nevertheless, the collected information can guide additional research to expand the acquired knowledge.

## 10. Conclusions

Irresponsible self-medication is a globally present problem with significant healthcare and socioeconomic impact, and managing it requires an integrated approach. It is important to recognize the necessity for responsible and informed self-medication, as well as possible threats of irresponsible self-medication in the population. Collaboration between healthcare professionals of different professions who provide direct care to patients is essential in educating patients on self-medication and in monitoring patients’ behaviors in this aspect. Restricted access to physicians due to various reasons can limit the needed frequency of regular check-ups, which can lead to increased self-medication. Subsequently, due to a lack of professional supervision, self-medication can easily slip into inappropriateness. Since access to community pharmacies without the need for a formal appointment is widespread and convenient for many patients, pharmacists as easily reachable HCPs could facilitate responsible self-medication practices by promoting appropriate drug use, adherence to treatment, and diminishing the misuse of drugs. Pharmacists should be more actively involved in screening possibly relevant ongoing self-medication and its prevention, as well as proactively providing counseling, especially in underserved communities. Reinforcement of cooperation between pharmacists, physicians, and patients may lead to more customized and effective management methods. This joint effort should set a path for enhancing health outcomes and decreasing healthcare costs. Ongoing surveillance of the medicines’ utilization and counseling of patients is important to public health systems in terms of both health and cost issues, which applies to both medically prescribed drugs and drugs that can be obtained without a prescription. In addition to the importance of the role of HCPs in supervising drug utilization and providing regular counseling, to minimize the risks of inappropriate self-medication, careful monitoring and valid regulations should be applied when obtaining medicines via online pharmacies.

Increased awareness of the various risks of inappropriate self-medication should be further expanded through customized public campaigns. Recommended interventions, such as educational schemes, public health awareness initiatives in diverse communities, and therapeutic training programs for various demographic groups, should be promptly implemented. Future studies should be conducted to gain deeper insights into the self-medication practices of different subpopulation groups to secure a better understanding of their motives and to ensure the development of more appropriate methods, as well as to assess the effectiveness of implemented interventions in achieving their goals, such as improving the level of knowledge and reducing irresponsible self-medication and its risks to make it a more appropriate part of self-care, as it should be.

## Figures and Tables

**Table 1:** Relevant studies related to self-medication in population.

Author(s) and Year	Country	Study Objectives and Design	Main Outcomes	Implications
Ylä-Rautio et al. (2020) [7]	Finland	- observational study that was conducted through a questionnaire in pharmacies	- significant proportion of the documented problems (26.3%) concerned high-risk OTC medications	Pharmaceutical advising should be accessible and actively provided for users to obtain safer self-medication.
Kłoda K et al. (2024) [25]	Poland	- online computer-assisted web interview for physicians to assess self-medication practices of adults and children	- the use of antibiotics by adults and children and the mental health of both populations seems to be particularly alarming	Educational and organizational support should be implemented at multiple levels.
Gebert et al. (2024) [26]	Germany	- a written questionnaire survey addressed younger and well-educated adults during public science events	- 59.3% of participants stated they regularly used self-medication in the last 12 months - the most common symptoms/complaints were headaches in 86.2% of respondents - as reason for making decision on self-medication, 92.6% of participants listed “intensity of complaints”	Practices to ensure availability of evidence-based information about self-medication in pharmacies should be expanded and scientifically researched to upgrade patient-related outcomes.
Barrenberg et al. (2018) [27]	Germany	- calculating seven-day prevalence of OTC drug use among adult population based on information provided by participants of the German Health Interview and Examination Survey for Adults	- the prevalence of OTC drug use in past seven days was 40.2% in total - OTC medicine use levels were higher than the self-medication prevalence found in the same data set, possibly because physicians often prescribe some OTC drugs	Prescription or recommendation of OTC drugs by physicians should be considered in future research and given justified scientific attention.
Klemenc-Ketis et al. (2010) [28]	Slovenia	- self-administered web-based questionnaire to assess the practice of self-treatment during the past year and compare healthcare and non-healthcare students	- the percentage of students, both healthcare and non-healthcare, who reported the use of some sort of self-medication during the study period was 92.3%	The level of awareness of risks of irresponsible medication should be lifted to a higher level through action and practices in education and public health actions.
Schmiedl et al. (2014) [15]	Germany	- observational multi-center study analyzed self-medication with over-the-counter drugs and self-medication-related adverse drug reactions leading to admissions in internal medicine departments of hospitals	- self-medication was implicated in 3.9% of patients with adverse drug reactions who were admitted to hospital - in 53.8% of these patients, ADRs were attributable to OTC drugs	The patients’ safety would be improved by development of programs focused on elderly patients and patients who receive prescribed medicines with interaction potential.
Kıroğlu et al. (2022) [29]	Turkey	- cross-sectional survey among outpatients on their first visit to the otorhinolaryngology department	- self-medication with conventional drugs before visiting a hospital was reported by 44.8% of respondents - the most frequently used drugs were medicine analgesics (31.7%) and antibiotics (21.9%).	To prevent inappropriate drug use regulations on conventional and herbal medicines usage should be put into practice.
Yeamans et al. (2024) [17]	European Union	- to examine prevalence and associated risks of self-medication among general population in European Union (EU) data from the third wave of the European Health Interview Surveys were utilized	- estimated prevalence of self-medication in EU was 34.3% - facilitators of self-medication were long-standing health problems, visits to physicians, and unmet needs for healthcare due to waiting lists or inability to afford medical examinations/treatment	Tools to find potentially dangerous self-medication practices and advise policy decisions adjacent to self-medication should be developed.
Lau et al. (1995) [32]	China	- students were interviewed to survey the practice of self-medication	- the reported rate of self-medication was 94.0% - most commonly used remedies were cough and cold preparations, antipyretics, and analgesics	The role of healthcare professionals should be more active to establish good self-care programs.
Martín-Pérez et al. (2016) [30]	Spain	- cross-sectional study to determine the prevalence and key drivers of parental administration of OTC drugs to children performed with data from the 2011 to 2012 Spanish National Health Survey among children	- 8.2% of analyzed children received OTC drugs in the 2 weeks prior to the survey - the most commonly used medicines were medicines for cold (25.5%), analgesics (30.3%), and antipyretics (22.8%)	Understanding factors that influence self-medication is important to recognize the parents who would be inclined toward it and to enhance the level of knowledge of responsible self-medication.
Jensen et al. (2014) [38]	Denmark	- quantitative cross-sectional survey was conducted on older children and their mothers - structured interviews with all children and self-report questionnaires for mothers regarding health, pain, and medicine use were used for data collection	- use of OTC analgesics in mothers was significantly associated with self-medication with OTC analgesics, specifically paracetamol in schoolchildren, even when the child’s pain was adjusted for (odds ratio 3.00, *p* = 0.008) - there was no clear connection found between child pain and the use of OTC analgesics	Information to parents about appropriate self-medication is important to increase awareness of responsible use of paracetamol in schoolchildren.
Du et al. (2009) [37]	Germany	- recording of all cases of drug use in a previous week among - children aged 0–17	- the finding was that 25.2% of participants had used self-medication in the previous week (17.0% used over-the-counter drugs, and 9.9% used other-source drugs) - the part of self-medication in total medicine use was 38.5%, and it included all medication classes	The shown behaviors represent justification for development and implementation of educational programs for parents.
Tarciuc et al. (2020) [36]	Romania	- pilot study that uses a questionnaire—investigation of the attitudes and the behaviors related to self-medication of a group of parents	- level of self-medication of parents who self-medicate their children was 70% - there was a significant relationship between parents’ beliefs about self-medication and their inclination to give medicines to their children without medical advice and between the probability of parental self-medication for their children and the number of diseases encountered by their children over the six months before the survey	Educational actions in the domain of responsible self-medication should be strengthened to prevent the risks associated with this practice.
Knopf et al. (2017) [39]	Germany	- study collected data on the use of medically prescribed and self-medicated medicines during the two weeks prior to the survey	- the reported rates of taking medicines prescribed by physicians was 58.9% for women and 52.0% for men, and rates for medicines that were not prescribed by physicians were 48.5% for women and 35.4% for men - the prevalence of the increase in prescribed medicines’ use was associated with age, and the prevalence of self-medication decreased with age	Ongoing surveillance of medicine consumption is particularly important to public health regarding health issues and costs.
Garofalo et al. (2015) [31]	Italy	- cross-sectional survey using a random sample of parents of students to register the prevalence, the key factors, and the causes of oral self-medication	- the prevalence of practiced self-medication at least once was 69.2% - the most frequently used medicines without prescription in previous year were non-steroidal anti-inflammatory drugs - inappropriate self-medication occurred in the last year at least once in two-thirds of respondents	A quite high frequency of oral self-medication that is inappropriate in majority of respondents warrants development and implementation of educational programs.
Gheorman et al. (2024) [40]	Romania	- study using questionnaire to pharmacists to evaluate the perceptions of pharmacists regarding self-medication and their role in managing self-medication and educating the public on rational use of medicines	- the frequency of self-medication and the perceived seriousness of conditions had a direct connection - self-medication was more habitual for minor disorders - education of population on the risks of self-medication significantly reduces its prevalence	It is necessary to have chosen informational campaigns and educational schemes for various demographic groups.
Ge et al. (2022) [35]	China	- study conducted using questionnaire to estimate self-medication status and associated factors	- the level of self-medication among adult participants was 99.06% - the most frequently purchased medicines were NSAIDs (58.57%) and vitamins/minerals (52.41%) - the rate of respondents who took into consideration the advice of medical staff when purchasing OTC medicines was 86.2%	Coordinated actions from regulators, healthcare professionals, and drug manufacturers should be implemented to influence and regulate self-medication in the population.
Figueiras et al. (2000) [41]	Spain	- cross-sectional study performed using a sample representative of the population of adults to identify the sociodemographic factors associated with self-medication and undesirable self-medication	- the prevalence of self-medication in two weeks prior to the interview was 12.7% - the reported prevalence of undesirable self-medication was 2.5%	There is a need for public health education programs aimed at improving the quality of self-medication behavior.
Niclós et al. (2017) [42]	Spain	- descriptive, cross-sectional study of the adult population to identify factors associated with self-medication	- the reported rate of using medications, both prescribed and non-prescribed, was 67% - reported rate of self-medication was 22.0%	Targeted health education on the risks of self-medication should be examined.
Lei et al. (2018) [18]	China	- residents were interviewed in the survey to collect information on self-medication behavior and associated factors	- almost half of respondents would select self-medication if they were ill, and 39.1% would see a physician - the most common diseases for which they practiced self-medication were cold and cough, cardiovascular disease, and gastrointestinal disease	Drug consultation services should be standardized to reduce the risk of inappropriate self-medication.
Okyay et al. (2017) [33]	Turkey	- cross-sectional study among university students via questionnaire on rational use of drugs	- the found prevalence of practicing self-medication in surveyed students was 63.4% - medicines that were most commonly self-medicated were analgesics (39.5), antibiotics (36.9%), and cold preparations (24.0%)	Educational activities should be implemented to avoid negative consequences of inappropriate self-medication
Meknassi Salime et al. (2025) [34]	Morocco	- a descriptive cross-sectional study was conducted through a questionnaire among parents	- reported prevalence of self-medication was 92.9% - the majority of parents self-medicated their children with antibiotics for transient fever, minimal pain, and nasopharyngitis - the most commonly used drugs were antipyretic analgesics and antibiotics	Therapeutic training programs planned for families should be developed in cooperation with healthcare professionals.

**Table 2:** Relevant studies related to self-medication during COVID-19 pandemic.

Author(s) and Year	Country	Study Objectives and Design	Main Outcomes	Implications
Karami et al. (2023) [48]	USA	- the assessment of monthly United States poison center data, including pediatric exposures to nonprescription paracetamol, ibuprofen, acetylsalicylic acid, and naproxen before and during the pandemic	- 75–90% of nonprescription analgesic/antipyretic exposures were single-substance - 84–92% of unintentional exposures involved children <6 year - intentional exposures mainly involved females (82–85%) and adolescents 13–17 years old (91–93%)	Findings highlight the importance of safely storing medications and being alert to signs that adolescents may need mental health support services.
Makowska et al. (2020) [43]	Poland	- online survey about participants’ experiences with self-medication during three-month COVID-19 lockdown using structured questionnaire	- 45.6% of respondents indicated that during the lockdown, they had engaged in inappropriate self-medication in at least one way - 16.6% self-medicated as a precaution, and 16.8% self-medicated prescription medicine without consultation	Appropriate public health programs should be developed to help people properly manage medications in situations where the availability of physicians is limited, such as lockdowns.
Aydın Aksoy et al. (2024) [47]	Turkey	- descriptive study to evaluate mothers’ use of nutrient supplements with OTC drugs for their children during COVID-19 pandemic	- 45.3% of mothers reported giving OTC medicines to their children - most frequently used were vitamin D, fish oil, multivitamins, vitamin C, immune boosters, zinc, probiotics, herbal teas, oral/nasal sprays, throat lozenges, and aspirin	Continued education of the public is relevant to positively influence maternal decision making concerning child health and medication practices.
King et al. (2022) [45]	USA	- two waves of interviews among participants who use drugs in nine prospective cohorts to estimate the prevalence and correlates of stockpiling drugs in the previous month during COVID-19 pandemic	- stockpiling of drugs was reported in 11.6% cases in the last month at baseline - stockpiling of drugs was significantly and positively associated with being greatly impacted by COVID-19 and with at least daily use of methamphetamine in the past month	Addressing the impact of COVID-19 on vulnerable people who use drugs possibly could help limit drug stockpiling, which may decrease rates of high-intensity stimulant use.
Chaudhry et al. (2022) [19]	Pakistan	- study performed using questionnaires aimed to assess the characteristics, practices, and associated factors of self-medication by the public during the COVID-19 pandemic	- among participants who were tested for COVID-19 during the pandemic, 34.3% engaged in self-medication - the most commonly used medicines and supplements were paracetamol (23.6%), azithromycin (14,9%), cough syrups (13%), OTC drugs, and oral vitamin supplements	Besides increasing awareness and understanding about the possible adverse effects of self-medication, partnerships with pharmacists must be developed and applied in order not to sell prescription medicines without a verified prescription.
Mejia et al. (2023) [49]	Latin America	- cross-sectional analytical study conducted through analysis of secondary data on the use of medicines and self-medication during COVID-19	- participants reported using paracetamol, ibuprofen, or antibiotics and disclosed this as self-medication in 26.9%, 16.6%, and 9.7% of cases, respectively - prevalence of self-medication in study population was significantly high, including the use of medicines not recommended for COVID-19 treatment and/or prevention	Public health measures should be implemented to fight against irresponsible self-medication to prevent negative impact on current health and future effectiveness of medicines, particularly in case of antibiotic self-medication.
Arboleda Forero et al. (2023) [52]	Columbia	- survey considering sociodemographic characteristics, self-medication with antibiotics, reasons for using these drugs, and types of antibiotics used in context of COVID-19	- self-medication with antibiotics was reported in 46% of cases - 47.4% of this population reported using antibiotics without medical prescription, usually for flu-like symptoms related to COVID-19	Increasing awareness of inappropriate self-medication with antibiotics and its possible consequences through targeted actions is important to ensure the controlled use of these medications.
Zhang et al. (2021) [51]	Australia	- an online survey was conducted at the height of the initial outbreak in the context of the COVID-19 pandemic in Australia	- 19.5% of participants took antibiotics to protect themselves from COVID-19 - there was a positive association between pandemic and self-medication - 35.6% of respondents used antibiotics for cold/flu - 23.2% of respondents used leftover antibiotics	Development and implementation of tailored education programs to raise awareness of risks of inappropriate self-medication is needed.
Mustafa et al. (2023) [54]	Pakistan	- descriptive cross-sectional study was conducted among healthcare professionals to assess their knowledge, attitude, and practices of self-medication during pandemic	- majority of participants had good knowledge and attitudes on self-medication, and 60% still practiced self-medication during COVID-19 pandemic - the most commonly used medicines were antipyretics (100%), antibiotics (80.4%), and vitamins (59.9%)	Increasing the awareness of appropriate use of antibiotics to contain development of antimicrobial resistance is necessary.
Salvador-Carrillo et al. (2024) [55]	Peru	- cross-sectional study was performed using e-survey to included population to describe the self-medication practices and associated risk factors during COVID-19 outbreak	- reported frequency of self-medication among participants was 35.93% - the most commonly used drugs without a prescription in self-medicated population were ivermectin (drops, 72.01%), paracetamol (41.24%), and azithromycin (25.81%)	There is a need to strengthen public health education, improve access to healthcare system, and reinforce education of HCPs to diminish self-medication practices, especially during disastrous events such as pandemic.
Stüdemann et al. (2024) [46]	Germany	- two questionnaires in an observational cohort study from positively tested COVID-19 outpatients to describe self-medication with OTC drugs and use of other remedies against symptoms of COVID-19	- the rate of participants who reported using medicines and other remedies to relieve COVID-19 symptoms was 74% - most frequently used medicines were ibuprofen (26%), paracetamol (21%), metamizole (14%), and acetylsalicylic acid (10%)	Further studies could be useful to examine a possible causal relationship between OTC medicines and COVID-19 disease course.
Jifar et al. (2024) [50]	Ethiopia	- community-based cross-sectional study was performed at selected drug retail outlets for community pharmacy clients during the COVID-19 pandemic to assess factors associated with self-medication	- the most frequently used medicines to treat or prevent COVID-19 were analgesics (42.4%), medicines for cold (29.5%), and antibiotics (26.7%) - the most common symptoms for the use of antibiotics were respiratory infection (14.3%), cough (5.8%), and sore throat (5.5%) - more than one-third of participants (36.6%) reported having medicines at home for treatment and prevention of COVID-19	Particular attention should be given to educate both HCPs and the public on the types of diseases that can be self-diagnosed and self-treated and the types of drugs to be used for self-medication.
Zheng et al. (2024) [57]	Macao	- face-to-face cross-sectional survey was performed to assess the self-medication patterns and intention to seek pharmacist advice among older adults during COVID-19 pandemic	- reported self-medication rate was 64.2% - the most commonly used medicines were over-the-counter and traditional medicines - majority of respondents engaged in self-medication to prevent or treat COVID-19 symptoms	Increasing the recognition of pharmacists as well as adapting pharmacy governance models and enhancing pharmacists’ self-perception should be addressed.
Souza et al. (2024) [56]	Brazil	- cross-sectional study conducted among students using an electronic questionnaire to evaluate prevalence and predictors of self-medication for COVID-19	- reported prevalence of self-medication was 14.9% - the main products used were ivermectin, vitamin C, vitamin D, tea, azithromycin, zinc, and propolis	Health education measures should be developed and implemented to reduce self-medication and guide the population regarding the risks of this practice.
Li et al. (2024) [53]	China	- cross-sectional study was conducted to estimate the prevalence of self-medication with antibiotics and associated factors in general public and HCPs during the COVID-19 pandemic	- the reported rate of self-medication with antibiotics was 10.25% in general population and 12.69% in HCPs	Efforts to increase awareness and promote the rational use of antibiotics require collective engagement.

**Table 3:** Relevant studies related to self-medication with antimicrobials (antibiotics).

Author(s) and Year	Country	Study Objectives and Design	Main Outcomes	Implications
Väänänen et al. (2006) [62]	Spain	- the study aimed to determine whether antibiotics are used for self-medication	- use of antibiotics was reported by 28% of the respondents during the previous 6 months—41% of antibiotic users bought antibiotics without a prescription - the most common indication for antibiotic use was common cold in 45% of cases, followed by sore throat in 17%	Importance of increasing AMR awareness and promoting rational and more supervised use of antibiotics to minimize the expansion of antibiotic resistance.
Grigoryan et al. (2006) [70]	Europe	- study surveyed the populations of 19 European countries to compare the prevalence of antimicrobial drug self-medication in previous year, intended self-medication and storage, and to identify the associated demographic characteristics	- the prevalences of actual self-medication and intended self-medication were high in Eastern and Southern Europe and low in Northern and Western Europe - the most common reasons for self-medication were throat symptoms - the main sources of medications were pharmacies and medication leftovers	Activities to diminish inappropriate self-medication should be directed toward prescribers, pharmacists, and patients.
Roberts et al. (2020) [73]	USA	- survey of prevalence and awareness of doctors of dental medicine of self-medication with non-prescribed antibiotics	- the rate of respondents who reported the use of antibiotics in the previous 2 years was 68.3% - approximately 1 in 16 surveyed older adults reported self-medication with non-prescribed antibiotics	There is a need for education on self-medication with antibiotics that were not prescribed to increase awareness, and it should be implemented in dental curricula.
Zhu et al. (2016) [67]	China	- online survey was conducted using questionnaire to evaluate self-medication practices	- 47.9% of participants reported a lifetime history of antibiotic self-medication - 65.9% of respondents had more than one episode of self-medication with antibiotics in the previous year - 73.5% of respondents used at least two different antibiotics	It is necessary to have firm regulations on antibiotic sales. In addition, public education supported by further healthcare reform is recommended.
Wang et al. (2018) [63]	China	- survey using self-administered online questionnaire among university students to evaluate the role of keeping antibiotics at home as driver of self-medication with antibiotics	- 29.6% of students self-medicated antibiotics when sick during the month before the survey - for students who reported keeping antibiotics at home, they were five times more likely to engage in self-medicating antibiotics when sick and 2.6 times more likely to self-medicate with antibiotics for prophylaxis than the other students	Findings about the practices of keeping antibiotics at home present a serious issue, which suggest there is a need for professional directives and population-tailored health education.
Bi et al. (2000) [64]	China	- study to assess self-medication and misuse of antibiotics in families using questionnaire for parents	- the reported rate of parents who self-medicated their children was 59.4% - approximately 51% of children had received self-medication from their parents on six or more occasions - rate of inappropriate antibiotics use was 35.7%	More education on proper antibiotic use and on possible risks associated with irresponsible self-medication of antibiotics is needed.
Napolitano et al. (2013) [72]	Italy	- cross-sectional survey using self-administered questionnaire conducted on a random sample of parents	- the rate of parents who were aware of the definition of antibiotic resistance was 9.8%, and for those who were aware of when it was appropriate to use antibiotics, it was 21.2% - the rate of participants who listed themselves as users of self-medication was 32.7%	Functional public health initiative should be implemented to ensure feasible and suitable mechanisms to change the practices of irresponsible self-medication.
Pedrolongo et al. (2024) [61]	Brazil	- online questionnaire on the consumption of antibiotics and NSAIDs by the adult population considering the 90 days prior to the survey	- the rate of respondents who used NSAIDs was 89.5%, and for antibiotics, it was 32.2%, regardless of whether or not these medications had been prescribed by doctors or dentists - the non-prescription use of antibiotics by the population was considered high, reaching one-third of the total number of volunteers who used these medicines	There is a need to strengthen the education of HCPs who prescribe and dispense antibiotics to contain self-medication to prevent even bigger scale of the problem.
Pei et al. (2023) [68]	China	- an online survey to evaluate the influence of uncertainty and negative emotions in parents’ self-medication of children with antibiotics	- the rate of parents engaged in self-medication with antibiotics to their children was 66.5% - uncertainty was positively associated with negative emotions, which were in turn positively associated with attitude toward self-medication with antibiotics	Understanding the psychological factors driving parental self-medication with antibiotics may inform customized interventions to promote responsible antibiotic use among parents.
Cruz et al. (2022) [74]	Colombia	- case–control study of factors connected to self-medication of antibiotics based on surveys of caregivers of pediatric patients brought to the emergency room with clinical symptoms suggestive of acute infection	- higher parental education was related to less self-medication - higher probability of self-medication was related to attitudes, such as always requesting antibiotics from their physicians, frequently purchasing antibiotics without a prescription, and giving advice on antibiotics among family members - knowledge about the risks of self-medication with antibiotics was low	These findings should inform future interventions to reduce self-medication in children.
Saif et al. (2024) [65]	Pakistan	- a cross-sectional quantitative study on knowledge, practices, attitude, and adherence to antibiotic therapy among adult population - individuals with poor knowledge were selected for video-based intervention programs - a post-intervention study was conducted to assess the improvement	- 39.2% of participants had self-medicated antibiotics in the previous 6 months - 42% of participants were non-adherent to antibiotic treatment plan - the most important barrier in adherence to antibiotic treatment was lack of proper information from HCPs - the postintervention part showed significant improvement in mean scores of knowledge, practices, attitude, and adherence related to antibiotics	Specific relevant measures are alarmingly needed to prevent antibiotics to become futile in eliminating various controllable microbial diseases.
Jifar et al. (2024) [50]	Ethiopia	- community-based cross-sectional study performed at selected drug retail outlets for community pharmacy clients during the COVID-19 pandemic to assess factors associated with self-medication	- the most frequently used medicines to treat or prevent COVID-19 were analgesics (42.4%), cold medicines (29.5%), and antibiotics (26.7%) - the most common symptoms of the use of antibiotics were respiratory infection (14.3%), cough (5.8%), and sore throat (5.5%) - more than one-third of participants (36.6%) reported having medicines at home for treatment and prevention of COVID-19	Particular attention should be given to educate both HCPs and the public on the types of diseases that can be self-diagnosed and self-treated and the types of drugs to be used for self-medication.
Nazari et al. (2024) [66]	Iran	- population-based cross-sectional study conducted and analyzed self-reported annual consumption of antibiotics, as well as a record of antibiotic use registered in insurance services	- reported annual prevalence of antibiotic self-medication was 30.3% - significant correlation was found between educational level and self-medication practices	It is necessary to increase the awareness of self-medication with antibiotics and AMR through development and implementation of inclusive public health strategies and coordinated efforts of HCPs.
Duan et al. (2024) [75]	China	- a systematic review was conducted and in-depth interviews to identify the key attributes of choices when parents self-medicate antibiotics for children’s upper respiratory tract infections	- symptom severity was the most important in parents’ decision making to self-medicate antibiotics, followed by the risk of side effects or resistance, duration, total cost, onset time of antibiotic, and antibiotic effectiveness	Sophisticated intervention strategy should be implemented to reinforce parents’ ability to differentiate mild from severe upper respiratory tract infections, as well as their knowledge of antibiotics.
Darakhvelidze et al. (2024) [76]	Georgia	- a cross-sectional study performed using self-administered online questionnaire to collect data on self-medication with antibiotics	- total prevalence of self-medication was 32.6% - the rate of self-medication of adults with antibiotics was 23.8% - -12.7% confirmed the self-medication of antibiotics without medical advice to treat minor family members	The level of knowledge of inappropriate self-medication with antibiotics should be increased, and the sales of antibiotics without physician’s prescription should be regulated.
Elhaddadi et al. (2024) [69]	Morocco	- a cross-sectional study conducted using a questionnaire-guided interview with parents to assess determinants of self-medication with antibiotics in pediatric population	- self-medication with antibiotics was reported in 68% of included families - main symptoms for self-medication with antibiotics were cough (43%) and fever (24%)	It is necessary to increase the level of knowledge on responsible use of antibiotics.

**Table 4:** Relevant studies related to self-medication in healthcare professionals or students of healthcare studies.

Author(s) and Year	Country	Study Objectives and Design	Main Outcomes	Implications
Aşut et al. (2025) [79]	Turkey	- descriptive cross-sectional study using anonymous online questionnaire to estimate psychiatrists’ and psychiatry residents’ knowledge and attitudes regarding self-medication	- the reported rate of self-medication in the previous year was 83% - the most common self-medicated medicines were antidepressants - many respondents were unaware of legal and ethical guidelines	There is a need for educational improvements and better support systems that could enhance help-seeking, which should finally result in better health outcomes for psychiatrist and psychiatry residents.
Balon (2007) [81]	USA	- survey based on mailed questionnaire asking whether the psychiatrist would or did self-treat for depression	- the rate of respondents who would consider self-medication or self-medicate if afflicted with mild/moderate depression was 43% - the rate of respondents who self-medicated themselves for depression in the past was 15.7%	A substantial number of psychiatrists would treat themselves for depression, perhaps because of fear of stigma, a permanent record, or other reasons.
Kim et al. (2025) [82]	South Korea	- data on self-prescription of physicians with opioids, sedative-hypnotics, and other psychotropic medications obtained from the Ministry of Food and Drug Safety	- about 7% of practicing physicians self-prescribed opioids, sedative-hypnotics, or other potentially habit-forming drugs	Since self-prescribing for some physicians could relate to use of more possibly habit-forming drugs than necessary, closer surveillance of such self-prescribing habits among physicians may be needed.
Rosvold et al. (2002) [97]	Norway	- mailed, anonymous questionnaire to evaluate behavior among physicians	- the percentage of respondents who performed self-treatment during the last three years was 75% - among participants who used prescription medicine, 73% were self-prescribing it - 13% of the physicians had negative experiences with self-treatment	Since many physicians practice self-medication when they are ill and may contact friends and colleagues when they need help from another physician, it is worth addressing situations like these during their education.
Benameur et al. (2019) [89]	Saudi Arabia	- cross-sectional study based on survey among healthcare students using questionnaires	- reported prevalence of self-medication with antibiotics was 58.4% - the most common symptom for self-medication with antibiotics was tonsillitis (54.1%) - the rate of awareness that self-medication with antibiotics is not safe and is inappropriate was 79.9%	Health education should be improved in the direction of addressing this issue in a way that would result in ameliorating students’ knowledge, awareness, and attitudes on the use of antibiotics.
Turan (2024) [90]	Turkey	- cross-sectional study with questionnaire on self-medication of medical students	- the rate of self-medication among participants was 96.1% - 19.3% of students reported using antibiotics without a prescription - keeping medicines at home/in the dormitory for later use and not having chronic disease were found to be effective factors in engaging in self-medication practices	Educational interventions to promote the development of responsible self-medication behaviors should be a part of medical school education to significantly enhance relevance of responsible practices of self-medication.
Galán Andrés et al. (2021) [88]	Spain	- descriptive study among nursing students to gain insight into self-medication among them	- the rate of respondents who reported using non-prescribed medicines in the previous month was 73.8% - most frequently used drugs off-prescription were analgesics in 88.91% of occasions - 53.2% of respondents obtained drugs without prescription from a home first aid kit	There is a need to review and improve education on rational medicines use and responsible self-medication in the curriculum.
Al Essa et al. (2019) [91]	Saudi Arabia	- cross-sectional study using an electronic questionnaire among health sciences students	- high prevalence of self-medication was reported (73.2%), with approximately 70% of respondents who reported that their drugs were not prescribed by physician - 21% reported that self-medication was their first method of treatment for all problems	It is necessary to educate students about responsible self-medication as well as the risks of irresponsible self-medication.
Alduraibi et al. (2022) [92]	Saudi Arabia	- cross-sectional study using questionnaire to estimate knowledge, attitudes, and practices of self-medication in medical and pharmacy students	- the majority (94.6%) of students had good knowledge of self-medication - 63.9% of the students reported practicing self- medication in the last 6 months - the most common medications used for self- medication were analgesics in 88.29% of cases	The importance of medical and pharmacy students as contributors to the public healthcare system and future HCPs should be reflected in proper education on responsible self-medication and good pharmacy practice.
Kokabisaghi et al. (2024) [96]	Iran	- descriptive–analytical cross-sectional study using online questionnaire among the students of health sciences programs, which evaluated the knowledge, attitude, and practice of self-medication during COVID-19	- the reported rate of self-medication among medical university students was 19% - the most common medication taken was paracetamol (69.01%) - the most common symptom causing participants to engage in self-medication was headache (67.36%)	Recognizing the future role of medical students, it is necessary to pay more attention to their education in this area, together with controlling the prevalence of self-medication.
Hassan et al. (2025) [93]	Egypt	- cross-sectional study carried out using questionnaire among undergraduate medical students to assess self-medication patterns	- the prevalence of self-medication was 71%, and 88.3% of respondents used pharmaceutical products - most common self-medicated drugs were analgesics (92.4%) - half of the students took antibiotics for self-medication	It is necessary to value diagnosis, awareness, and seriousness of self-medication.
Jayawardhana et al. (2023) [94]	Sri Lanka	- cross-sectional study conducted using questionnaire among medical students to explore key factors that promote antibiotic use and misuse amongst medical students	- 46.4% of participants reported the use of antibiotics without a prescription - 32.0% reported keeping leftover antibiotics for future use; 23.3%—not completing the course of antibiotics, 21.3%—use of leftover antibiotics	Although students advanced considering knowledge, the self-medication practices remain the same over the years, which revealed the need to identify the additional drivers of antibiotic misuse among medical undergraduates.

**Table 5:** Relevant studies related to self-medication and storing medicines at home.

Author(s) and Year	Country	Study Objectives and Design	Main Outcomes	Implications
Louhisalmi et al. (2025) [105]	Finland	- the online survey conducted among customers of University Pharmacy to determine the amount, type, and storage practices of drugs in households	- 40% of drugs were to be stored safely - the main reasons for keeping unnecessary or expired medicines were improved health (39.2%), medication changes (31.9%), and oversized packs (28.0%)	The study showed that households had many medicines, highlighting the need for better storage and optimized packaging to improve safety, reduce waste, and enhance rational pharmacotherapy.
Tieu Mai Diep et al. (2024) [102]	Vietnam	- community-based cross-sectional study was conducted to research the prevalence of and identify the factors that influence medicine storage and medicine storage practice	- drugs were found in 71.6% of surveyed households - the most common type of medicines were analgesics - 68.1% of households stored drugs for future use - 9.4% of households did not keep medicine in the appropriate packaging - 19.4% of households did not check the expiry date of their medicine	Considering the high prevalence of household medication storage and some inappropriate storage behaviors, attention should be given to the development of effective interventions and policies to promote safe and appropriate storage practices.
Volkos et al. (2024) [107]	Greece	- cross-sectional study during regular appointments to assess the prevalence of self-medication of psychotropic medicines and supplements without medical advice, including home storage	- 38.4% of respondents reported having psychotropic medicines stored in home medical cabinets - 27.4% practiced self-medication with psychotropic medicines in the last 12 months	The findings could potentially inform primary care providers to focus on patients most likely to be users of psychotropic medicines without medical advice.
Köksoy (2024) [103]	Turkey	- cross-sectional study to identify unused pharmaceuticals, determine the number of unused medicines, and analyze practices of disposal methods	- Findings of first sample group: - drugs included drugs kept for reuse (41%) and those disposed of in household garbage (81%) - the most frequently unused pharmaceuticals were paracetamol, preparations for cold, dexketoprofen, diclofenac, amoxicillin, and betalactamase inhibitor - self-medication of unused medicines at home was reported in 94.1% of cases - Findings of second sample group: 50.6% of found drugs were used, and 49.4% were found to be unused	There is an obvious need for increasing public awareness on inappropriateness of practices of storing unused medicines, as well as expired and improperly stored medicines, and giving directions toward responsible medicine use and disposal.
Beuscart et al. (2019) [106]	France	- study conducted through interviews with older adults (≥65) taking at least five medications daily - it was also offered to examine the patient’s home medicine cabinet	- adverse events were reported in 42.0% of participants; 27.6% of participants reported difficulties in preparing, administering, and/or swallowing medications - medication storage problems were found in 21.7% and expired drugs in 40.7% of cases - potentially inappropriate medications were found in 15.0%, and redundant medications were found in 20.4% of cases	Pharmacists working in community pharmacies could identify high-priority older patients that need help with managing medications by asking simple questions about difficulties in managing, administering, taking, or storing medications.
De Bolle et al. (2008) [101]	Belgium	- cross-sectional study conducted in community pharmacies, and adult pharmacy customers were visited in their homes by pharmacy students, where home medical cabinets were inspected	- in 1/3 of the households, drugs were not stored safely - the percentage of prescription drugs was 34% - the most frequently found medicines were nonopioid analgesics (7.2%), NSAIDs (6.9%), nasal decongestants (3.5%), and antinausea agents (3.2%) - percentage of found expired drugs was 9% - the rate of self-medication was 56% for all drugs (OTC drugs—74%; prescription drugs—21%)	Since many younger participants showed inclination toward self-medication, there is a justified need to develop awareness of the risks of self-medication to avoid the risks connected with it.
Tourinho et al. (2008) [104]	Brazil	- descriptive population study with minor participants based on a home survey of a simple random sample - participants were divided into 2 groups based on medication use - medical prescription and self-medication (lay advice)	- 79.6% of total medicines found in households were pharmaceutical preparations - 76.5% of medicines were in cardboard boxes and within easy reach of children aged ≤ 6 years - the most frequently used drugs were analgesics/antipyretics (26.8%) and systemic antibiotics (15.3%) - significantly larger hoards of these drugs were found in self-medication group	To encourage rational use, minimize waste, and stimulate safe storage of drugs, it is necessary to develop and implement awareness campaigns.
Aljinović-Vučić et al. (2005) [95]	Croatia	- cross-sectional questionnaire-based survey to assess the content of household drug supplies and self-medication practices of healthcare students who inventoried medical cabinets in their family households and interviewed the household members on drug keeping and self-medication practice	- drugs past expiry dates and/or with purpose unknown to the household members were reported in 27% of the households - drugs most frequently found in households were NSAIDs (97%), followed by antibiotics (46%) - in households where they were found, NSAIDs were self-medicated in 88% of cases, and self-medication of antibiotics was practiced in 37% of them	There is a need to increase awareness on responsible self-medication and enhance education on irresponsible self-medication of antibiotics.

**Table 6:** Summary of practical recommendations.

Recommendations
**General**
Educational and organizational support to understand the risks of inappropriate self-medication and to prevent consequences associated with it should be implemented at multiple levels: social (information campaigns and educational institutions), the healthcare system (increasing the role of medical professionals), and in the legislative area (advise policy decisions related to self-medication and regulators’ participation).
**Social**
Develop and implement educational activities and functional public health initiatives through the following: - Tailored informational campaigns, educational schemes, and therapeutic training programs for various demographic groups in cooperation with healthcare professionals to reinforce the safety of patients, especially of more vulnerable family members (children, adolescents, and the elderly), by motivating more responsible medical practices within homes; - Understanding factors that influence self-medication to recognize patients who would be inclined toward it; - Education of both HCPs and the public on the types of diseases that can be self-diagnosed and self-treated, together with education on the types of drugs to be used for self-medication and drugs with interactive potential; - Development of appropriate public health programs with the aim of helping people to acquire reliable health information and guidance on how to properly manage their medication in situations where the availability of HCPs is limited, such as during health crises like the pandemic; - Development of easily accessible digital health tools, which include reliable information prepared by HCPs to assist with self-management of acute and chronic conditions
**Health systems and healthcare professionals**
Healthcare professionals should assume a more active role through the following actions: - Strengthened partnership between HCPs (especially family physicians and pharmacists) to identify high-priority patients who need help with managing medications and/or are inclined toward self-medication and recognizing in a timely manner when patients should be referred to physicians, which could avoid the costs of additional medical treatments; - Attempts to discover a possibility of relevant ongoing self-medication, as well as perform medication reviews to discover possibly improper medication practices so that more thorough instructions on given medicines and their proper use could be provided; - Practices that warrant evidence-based information on self-medication in pharmacies, such as pharmaceutical advising and drug consultation services that should be accessible, standardized, and actively provided for users to get safer self-medication and upgrade patient-related outcomes; - Revision of current status and implementation of actions to regulate access to medications via online pharmacies; - Coordinated actions from HCPs and drug manufacturers, which should be developed and implemented to influence and regulate self-medication of population.
**Antimicrobials/antibiotics**
Efforts should be made to increase the awareness of the risks of inappropriate self-medication and promote the rational use of antibiotics to prevent an even bigger scale of antimicrobial resistance development, which requires coordinated efforts of HCPs and collective engagement through the following actions: - More targeted educational activities to diminish irresponsible antibiotic use, such as inclusive public health strategies, which should be directed toward prescribers, pharmacists, and patients; - Strengthening the education of HCPs who prescribe and dispense antibiotics; - Developing sophisticated intervention strategies to reinforce patients’ ability to differentiate mild from severe upper respiratory tract infections, as well as their knowledge of antibiotics; - Implementing professional directives and population-tailored health education directed against inappropriate practices of keeping antibiotics at home to contain possible risks.
**Education of healthcare professionals (current and future)**
HCP education should be improved to address the issue of self-medication/self-prescribing and rational medicines use in a way that would result in improving students’ knowledge, awareness, and attitudes on the subject through the following actions: - Recognizing the role of medical and pharmacy students in self-medication as future HCPs and their value as contributors to the public healthcare system should be reflected in the curriculum; - Creating educational interventions to promote responsible self-medication, including antibiotics, which should be a part of healthcare education beginning in the first year to enhance responsible practices.
**Home pharmacies/Medicine cabinets**
To encourage rational use, stimulate the safe storage of drugs, and minimize waste, it is necessary to develop and implement public awareness campaigns on the possible risks of storing unused medicines, as well as expired and improperly stored medicines, and give clear directions regarding responsible medicine use and disposal through the following actions: - Develop effective interventions and policies to promote safe and appropriate drug storage practices; - Optimize packaging to improve safety, reduce waste, and enhance rational pharmacotherapy; - Provide general guidance to patients to periodically check the contents of the medicines’ cabinets, read package leaflets, check expiry dates, and safely discard all medicines past their due date.

## Data Availability

Data sharing is not applicable to this article.

## Supplementary Materials

The following supporting information can be downloaded at: https://www.mdpi.com/article/10.3390/healthcare13151872/s1, Table S1. Overview of features of studies related to self-medication in population. Table S2. Overview of features of studies related to self-medication during COVID-19 pandemic. Table S3. Overview of features of studies related to self-medication with antimicrobials (antibiotics). Table S4. Overview of features of studies related to self-medication in health care professionals or students of healthcare studies. Table S5. Overview of features of studies related to self-medication and storing medicines at home.

## Abbreviations

The following abbreviations are used in this manuscript:

WHO	World Health Organization
WMA	World Medical Association
HCP	Healthcare Professional
ADR	Adverse Drug Reaction
OTC	Over The Counter
COVID-19	Coronavirus Disease 2019
NSAID	Non-Steroidal Anti-inflammatory Drug
