# Supplementary material for: Self-Medication as a Global Health Concern: Overview of Practices and Associated Factors—A Narrative Review
Source: Healthcare (Basel). 2025 Jul 31;13(15):1872. doi: 10.3390/healthcare13151872 (PMC12346020; doi:10.3390/healthcare13151872)
Supplement: Supplementary file 1 [file healthcare-13-01872-s001.zip › healthcare-3767575-supplementary.pdf]

Supplementary Materials

Table S1. Overview of features of studies related to self-medication in population

Author(s) and Year	Study Title	Study Objectives and Design	Main outcomes	Implications
Ylä-Rautio et al. (2020) [7]	Drug-related problems and pharmacy interventions in non-prescription medication, with a focus on high-risk over-the-counter medications	- observational study that was conducted though a questionnaire<br>- pharmacists registered the noticed drug-related problems and pharmacy interventions in over-the-counter medication for 1 week using an electronic study form	- 52 community pharmacies documented 339 drug-related problems in 0.6% of over-the-counter customers<br>- "Uncertainty about the indication for the drug" was the most common problem reported in 39.2% of cases<br>- a significant percentage of the registered problems was related to high-risk over-the-counter medications (26.3%)<br>- majority of these cases were associated with non-steroidal anti-inflammatory drugs (21.8%)<br>- pharmacist's intervention involved counselling for majority of drug-related problems (87%)<br>- the pharmacy intervention was precautionary in more than half of the problem cases	As the choice and utilization of over-the-counter medications continuously expands, pharmaceutical advising should be accessible and actively provided for users to obtain safer self-medication.
Kłoda K et al. (2024) [25]	Self-medication of adults and children in Poland - results from outpatient health care physicians' online questionnaire	- online computer-assisted web interview that was addressed to outpatient healthcare physicians to assess self-medication practices of adults and children in Poland	- perspective of physicians for the main three reasons for choosing self-medication in Poland were: taking advice from other people - family members or friends (59.1%), finding information regarding treatment online (52.9%) and ability to self-medicate this kind of symptoms/disease (51.6%)<br>- for adult patients an independent decision to start antibiotic therapy was made in 72.1% of cases,<br>- for pediatric patients, the decision to start treatment with antibiotics was made in 39.8% of cases<br>- custodians of children patients were more likely to visit the physician immediately with symptoms than in the case of adult patients (42.2% vs. 22.1%, $p < 0.001$).	There is a need for educational and organizational support in segment of self-medication at multiple levels - social (information campaigns, school education), the healthcare system (increasing the role of medical professionals, including pharmacists), and in the legislative area.

			- the use of antibiotics by adults and children and the mental health of both populations seems to be particularly alarming	
Gebert et al. (2024) [26]	What Do Younger and Well-Educated Adults Think about Self-Medication? Results of a Survey during a Public Science Event at Leipzig University	- a written questionnaire survey addressed the frequency of self-medication use in the last 12 months and attitudes and opinions on self-medication of younger and well-educated adults	- 59.3% of participants stated they regularly used self-medication in the last 12 months<br>- the most common symptoms/complaints were headaches in 86.2% of respondents<br>- preconditions were mild complaints/symptoms in 94.7%<br>- as limitation of self-medication, duration was listed in 91.6% cases<br>- the main identified risk was "self-medication may cause adverse drug reactions" reported by 94.2% respondents<br>- the main identified fear was "developing a habituation effect" reported by 58.7% respondents<br>- the main information sources on adequate medication were pharmacists in 93.7% of cases<br>- as main factor that influenced choice of drug for self-medication majority respondents listed "physician's recommendation" in 89.3%<br>- as reason for making decision on self-medication 92.6% of participants listed "intensity of complaints", 92.6%<br>- 61.3% believed that they could choose appropriate self-medication	Even schooled patients need professional empirical care from community pharmacies. Practices that warrant evidence-based information about self-medication in pharmacies should be expanded and scientifically researched to upgrade patients related outcomes. Recognizing in a timely manner when patients should be referred to physicians is also important and could save the costs of medical treatment.
Barrenberg et al. (2018) [27]	Over-The-Counter (OTC) Drug Consumption among Adults Living in Germany: Results from the German Health Interview and Examination Survey for Adults 2008–2011 (DEGS1)	- calculating seven-day prevalence of OTC drug use among adult population based on information provided by participants of the German Health Interview and Examination Survey for Adults (DEGS1)	- the prevalence of OTC drug use in past seven days was 40.2% in total<br>- it was higher in women (47.16%) than in men (33.17%)<br>- factors identified as forecasters of use of OTC medicines were female gender, > 60 years, reduced health status, Rx drug use, and multi-morbidity<br>- OTC medicines use levels were higher than the self-medication prevalence found in the same data set possibly because physicians often prescribe some OTC drugs	Since considerable amount of OTC drugs can be recommended or prescribed by physicians, this should be considered in future research on these topics and given justified scientific attention.

Klemenc-Ketis et al. (2010) [28]	Self-medication among healthcare and non-healthcare students at University of Ljubljana, Slovenia	- self-administered web-based questionnaire<br>- assessing the practice of self-treatment during the past year and comparing healthcare and non-healthcare students	- the percentage of students, both healthcare and non-healthcare, who reported the use of some sort of self-medication during the study period was 92.3%<br>- self-medication was practiced in more healthcare students in their senior year (94.1%) than those in their junior year (89.4%) ($p = 0.04$)<br>- the opinion of healthcare students was that self-medication without improvement of the symptoms should last for 1 week or less ($p = 0.05$)<br>- healthcare students acquired medicines for self-medication from pharmacies,<br>- healthcare students thought that previous doctors' advice in a similar situation was a more important reason for self-medication, would seek the advice of a physician or pharmacist for different ways of self-treatment, and thought that self-medication was not very safe<br>- non-healthcare students acquired the drugs from healers and friends	The level of awareness of responsible self-medication and risks of irresponsible medication should be lifted to a higher level through action and practices in education and public health actions.
Schmiedl et al. (2014) [15]	Self-medication with over-the-counter and prescribed drugs causing adverse-drug-reaction-related hospital admissions: results of a prospective, long-term multi-centre study	- observational multi-center study analyzed self-medication with over-the-counter drugs and self-medication related adverse drug reactions leading to admissions in internal medicine departments of hospitals	- self-medication was implicated in 3.9% of patients with adverse drug reactions who were admitted to hospital<br>- in 53.8 % of these patients ADRs were attributable to OTC drugs<br>- remaining ADRs were due to formerly prescribed drugs and potential OTC drugs<br>- ADRs related to self-medication were mostly gastrointestinal disorders caused by non-steroidal anti-inflammatory drugs (most frequently OTC acetylsalicylic acid (ASA)<br>- 38.3 % of the patients with ADRs related to self-medication relevant drug-drug interaction was reported between a self-medication and a prescribed medication (most frequently between ASA taken as	The patients' safety would be improved by development of programs focused on elderly patients and patients who receive prescribed medicines with interaction potential.

			an OTC drug and prescribed diclofenac)	
Kıroğlu et al. (2022) [29]	Self-medication practices with conventional and herbal drugs among ear, nose, and throat patients	- cross-sectional survey was carried out among all ear, nose, and throat outpatients on their first visit to the otorhinolaryngology department at a tertiary care hospital to evaluate their practices of self-medication with conventional and herbal drugs	- self-medication with conventional drugs before visiting a hospital was reported by 44.8% of respondents<br>- the most frequently used drugs were medicine analgesics (31.7%) and antibiotics (21.9%).<br>- at least one herbal drug was used by almost half of the patients (49.2%)<br>- participants perceived that herbal drugs are effective for ear, nose, and throat problems in 36.6% of cases<br>- patients were not aware of herbal-drug interaction with other medications in 22.9% of cases	To prevent inappropriate drug use regulations on conventional and herbal medicines usage should be put into a practice.
Yeamans et al. (2024) [17]	Self-medication among general population in the European Union: prevalence and associated factors	- to examine prevalence and associated risks of self-medication among general population in European Union (EU) data from the third wave of the European Health Interview Surveys were utilized	- estimated prevalence of self-medication in EU was 34.3%<br>- parameters associated with greater self-medications prevalence were age 25-44 (versus ages 75+), female gender, immigrants born in other EU countries, living in the cities<br>- facilitators of self-medication were long-standing health problems, visits to physicians (both general practitioners and specialists) and unmet needs for healthcare due to waiting lists or inability to afford medical examinations/treatment<br>- parameters found to be associated with self-medication were smoking, vaping, drinking alcohol, and higher levels of physical activity	There is a need to raise awareness of potential risks of irresponsible self-medication and need to develop tools to find potentially dangerous self-medication practices and advise policy decisions adjacent to self-medication.
Lau et al. (1995) [32]	Self-medication among university students in Hong Kong	- students were interviewed to survey the practice of self-medication	- the reported rate of self-medication was 94.0%<br>- most used remedies were cough and cold preparations, antipyretics and analgesics<br>- self-medicated drugs were mostly obtained from home medicine cabinets and pharmacy shops (which were not necessarily operated by registered pharmacists)	The role of healthcare professionals should be more active to establish good self-care programs.

			- the source of information on the medications participants used were family members and previous illness experience<br>- the healthcare professionals had only minor role in providing drug information	
Martín-Pérez et al. (2016) [30]	Parental administration of over-the-counter medication to Spanish children: A population-based national study	-cross-sectional study to determine the prevalence and key drivers of parental administration of over-the-counter (OTC) drugs to children in Spain performed with data from the 2011 to 2012 Spanish National Health Survey among children (birth to 14 years old)	- 8.2% of analyzed children received OTC drugs in the 2 weeks prior to the survey<br>- the most used medicines were medicines for cold (25.5%), analgesics (30.3%), and antipyretics (22.8%)<br>- variables that predict parental administration of OTC drugs included older age groups (10- to 15-year-olds), no chronic illness, children with limitations of normal activity, parents of middle and higher social status, and completion of secondary school or higher education	It is important to understand factors that influence self-medication to recognize the parents who would be inclined towards it and ensure that they are aware of the meaning of appropriate self-medication and how to provide it to children as well as to understand the possible risks if self-medication is inappropriate.
Jensen et al. (2014) [38]	Association of maternal self-medication and over-the-counter analgesics for children	- quantitative cross-sectional survey was conducted on children aged 6 to 11 years and their mothers in the framework of the Demonstration Of A Study To Coordinate And Perform Human Biomonitoring On A European Scale (DEMOCOPHES) European project to evaluate the association of maternal self-medication and self-medication of children of school age with OTC analgesics<br>- structured interviews with all children and self-report questionnaires for mothers regarding health, pain, and medicine use were used for data collection	- use of OTC analgesics in mothers was significantly associated with self-medication with OTC analgesics, specifically paracetamol in schoolchildren, even when the child's pain was adjusted for (odds ratio 3.00, P = .008)<br>- there was not a clear connection found between child pain and the use of OTC analgesic<br>- maternal health (self-rated health, chronic pain, chronic disease, daily medicine intake) did not have significant influence on child use of OTC analgesics<br>- maternal health seems to be of less importance	Information to parents about appropriate self-medication is important to increase awareness of responsible use of paracetamol in schoolchildren.

Tarciuc et al. (2020) [36]	Patterns and Factors Associated with Self-Medication among the Pediatric Population in Romania	- pilot study that uses a questionnaire - investigation of the attitudes and the behaviors related to self-medication of a group of parents in relation to their beliefs and perceived risks of the administration of medicine to their children without medical advice, prevalence of self-medication, symptoms, and types of drugs most used without medical advice	- level of self-medication of parents who self-medicate their children was 70%<br>- there was a significant relation between parents' beliefs about self-medication and their inclination to give medicines to their children without medical advice and between the probability of parental self-medication for their children and the number of diseases encountered by their children over the six months before the survey<br>- when parents had appropriate understanding of self-medication risks, it was not aligned with actual behavior therefore resulting in parents continued to administer drugs to their children without medical advice	Educational actions in the domain of responsible self-medication should be strengthened to prevent the risks associated with this practice.
Knopf et al. (2017) [39]	Use of medicines by adults in Germany	- surveys that map the population's use of medicines<br>- study collected data on the use of medically prescribed and self-medicated medicines during the two weeks prior to the survey	- the reported rate of medicines prescribed by physicians was 58.9% for women and 52.0% for men<br>- the reported rate of taking medicines that were not prescribed by physicians were 48.5% for women and 35.4% for men<br>- the prevalence of the increase in prescribed medicines' use was associated with age<br>- the prevalence of self-medication decreased with age<br>- regarding educational status, prevalence rates of self-medication were higher for the upper educational group than for the lower educational group	Ongoing surveillance of medicines consumption is particularly important to public health regarding health issues as well as costs.
Du et al. (2009) [37]	Self-medication among children and adolescents in Germany: results of the National Health Survey for Children and Adolescents (KiGGS)	- recording of all cases of drugs use in a previous week among children aged 0-17 years who participated in the 2003-2006 German Health Interview and Examination Survey for Children and Adolescents	- the finding was that 25.2% of participants had used self-medication in the previous week (17.0% used over-the-counter drugs and 9.9% other-sources drugs)<br>- the part of self-medication in total medicine use was 38.5% and it included all medication classes<br>- the most used medicines were ones acting on the respiratory	Behaviors related to self-medication in younger children using cough and cold medicines and aspirin suggested irresponsible drug use which can be connected to serious risks. These behaviors should be closely surveilled and represent justification for development and

			system (32.1%), gastrointestinal tract and metabolism (21.6%), skin (14.2%) and nervous system (11.3%), and homoeopathic preparations (8.6%)<br>- overall use of aspirin among children <12 years old was 0.3%<br>- use of cough and cold medicines was 4.4%, especially among children < 6 years old<br>- there was close association of self-medication to older adolescent ages of between 14 and 17 years, children with a poor health status, with no immigration background, from families with a higher household income and with mothers with a higher educational level	implementation of educational programs for parents.
Garofalo et al. (2015) [31]	Self-medication practices among parents in Italy	- cross-sectional survey using a random sample of parents of students to register the prevalence, the key factors, and the causes of oral medication use without the prescription of a physician, among a random sample	- the level of practiced self-medication at least once was 69.2%<br>- higher probability for engaging in self-medication were associated with female gender, younger age, and those who had a health problem in the previous year<br>- lower probability for engaging in self-medication were found in participants with a middle or lower school level of education<br>- among participants who reported engagement of self-medication, 53.4% have practiced at least once in the previous year and this behavior was more probable for those who have had a health problem<br>- the most frequently used medicines without prescription in previous year were nonsteroidal anti-inflammatory drugs<br>- inappropriate self-medication occurred in the last year at least once in two-thirds of respondents<br>- among participants who did not report a self-medication, 13.1% were prepared to practice it	A quite high frequency of oral self-medication which is inappropriate in majority of cases has a potential to negatively impact health status which warrants development and implementation of educational programs.

			- female gender was associated with higher inclination to engage in self-medication<br>- a secondary school level of education was associated with a lower willingness to engage in self-medication	
Gheorman et al. (2024) [40]	A Multicenter Survey on Pharmacists' Perspectives on Self-Medication Issues in Romania: A Descriptive Study Towards Sustainable and Safe Pharmaceutical Practices	- study conducted administering a questionnaire to pharmacists from various regions of Romania to evaluate the perceptions of pharmacists regarding self-medication and their role in managing self-medication and educating the public on rational use of medicines	- the frequency of self-medication and the perceived seriousness of conditions were in a direct connection<br>- self-medication was more habitual for minor disorders<br>- it is less probable that experienced pharmacists would encourage self-medication<br>- education of population on the risks of self-medication significantly reduces its prevalence	It is necessary to have chosen informational campaigns and educational schemes for various demographic groups.
Ge et al. (2022) [35]	Self-medication in Chinese residents and the related factors of whether or not they would take suggestions from medical staff as an important consideration during self-medication	- study performed using questionnaire that was developed to estimate self-medication status and associated factors, and important considerations in decision if Chinese residents would take into account HCPs suggestions during self-medication	- the level of self-medication among adult participants was 99.06%<br>- the most purchased medicines were NSAIDs (58.57%), and vitamins/minerals (52.41%)<br>- the rate of respondents who took into consideration the advice of medical staff when purchasing OTC medicines was 86.2%<br>- other important considerations when purchasing OTC medicines were drug safety and drug efficacy<br>- extremely high level of self-medication among respondents could be attributed to the moment when the study was carried out – immediately after break of COVID-19	Coordinated actions from regulators, healthcare professionals and drug manufacturers should be implemented to influence and regulate self-medication of population.
Figueiras et al. (2000) [41]	Sociodemographic factors related to self-medication in Spain	- a cross-sectional study was performed using a sample representative of the population of adults of ≥ 16 years to identify the sociodemographic factors associated to self-medication and undesirable self-medication	- the prevalence rate of self-medication in two weeks prior to the interview was 12.7%<br>- parameters associated with higher prevalence of self-medication were female gender, living alone, living in large cities<br>- among respondents who had acute disorders, the prevalence of self-medication was higher for	There is a need for public health education programs aimed at improving the quality of self-medication behavior.

			those with higher educational levels<br>- the reported prevalence of undesirable self-medication was 2.5%<br>- parameters associated with undesirable self-medication were > 40 years (compared to < 27 years), living alone and in students (compared with full time employees)	
Niclós et al. (2017) [42]	Factors associated with self-medication in Spain: a cross-sectional study in different age groups	- descriptive, cross-sectional study of the adult population (≥ 16 years), using data from the 2009 European Health Interview Survey to identify factors associated with self-medication in Spain	- the reported rate of using medications, both prescribed and non-prescribed, was 67%<br>- reported rate of self-medication was 22.0%<br>- variables strongly associated with self-medication were gender, educational level, age, chronic illness and physical activity	The stricter regulation on over-the-counter medicines may be needed to diminish health risks related to self-medication. Targeted health education on the risks of self-medication should be examined.
Lei et al. (2018) [18]	Self-Medication Practice and Associated Factors among Residents in Wuhan, China	- residents were interviewed from randomly selected four districts in China in the survey to collect information of self-medication behavior and associated factors	- almost half of respondents would select self-medication if they were ill, and 39.1% would see a physician<br>- the most common diseases for which respondents used self-medication were cold and cough, cardiovascular disease and gastrointestinal disease<br>- main reason for self-medication was perception that disease was not severe (enough) to visit a physician in 45% of cases, respondents didn't think that the bothering of visiting physician was worth the effort (23%), respondents didn't want to pay high medical costs (15%), respondents didn't have time to visit physician (12%)	Regulators should strengthen instructions on the use of medicines and pharmacists should actively advise patients on the medicines they use. Drug consultation services should be standardized to reduce the risk of inappropriate self-medication.
Okyay et al. (2017) [33]	Self-medication practices and rational drug use habits among university students: a cross-sectional study from Kahramanmaraş, Turkey	- cross-sectional study was conducted among university students who filled out a questionnaire on rational use of drugs to estimate practices of self-medication and rational drug use	- the found prevalence of practicing self-medication in surveyed students was 63.4%<br>- medicines that were most commonly self-medicated were analgesics (39.5), antibiotics (36.9%) and cold preparations (24.0%)<br>- the rate of respondents who stated they were familiar with	To avoid negative consequences of inappropriate self-medication educational activities should be implemented with the cooperation of healthcare professionals, educational institutions, non-governmental

			rational use of drugs and rational use of antibiotics was 45.9%<br>- among students who stated they were familiar with rational use of drugs levels rates of reading/checking the instructions in leaflet, understanding the context of the leaflet, compliance with the duration of antibiotic treatment and consulting a physician in case of a side effect were significantly higher than among those who weren't familiar with rational use of drugs	organizations and media.
Meknassi Salime et al. (2025) [34]	Self-medication of the pediatric population by parents in Morocco: Survey in the Midelt region	- a descriptive cross-sectional study was conducted through a questionnaire among parents of children under 12 years old visiting pharmacies to evaluate the prevalence of children's self-medication by parents	- reported prevalence of self-medication was 92.9%<br>- the majority of parents self-medicated their children with antibiotics for transient fever, minimal pain and nasopharyngitis<br>- self-medication starts between 6 months and 2 years - 41.5% of parents used age as a norm to determine the dose, 49.2% exchanged the dose measurement system between two medications, 76.3% used drug combinations for self-medication<br>- the most commonly used drugs were antipyretic analgesics and antibiotics<br>- 42.2% use the syrup or oral suspension form when self-medicating their children<br>- 64% reported that their source of information relating to drugs is pharmacist	There is a necessity to develop a therapeutic training program planned for families, in cooperation with healthcare professionals to reinforce the safety of children by motivating more responsible medical practices within homes.

Table S2. Overview of features of studies related to self-medication during COVID-19 pandemic

Author(s) and Year	Study Title	Study Objectives and Design	Main outcomes	Implications
Karami et al. (2023) [48]	Trends in pediatric nonprescription analgesic/antipyretic exposures during the COVID-19 pandemic	- the assessment of monthly United States poison center data including pediatric exposures to nonprescription paracetamol, ibuprofen, acetylsalicylic acid,	- 75–90% nonprescription analgesic/antipyretic exposures were single-substance<br>- 84–92% of unintentional exposures involved children <6 year<br>- intentional exposures involved mainly females	Findings highlight the importance of safely storing medications and being alert to signs that adolescents may need mental health support services

		and naproxen before and during the pandemic	(82–85%) and adolescents,13–17 years (91–93%)<br>- most intentional exposures were classified as suspected suicide.<br>- unintentional exposures declined for all four analgesics/antipyretics among children < 6 years immediately after the WHO declared COVID-19 a pandemic<br>- most significant decline was found for ibuprofen (30–39%)<br>- this decrease was accredited to an acute cut in influenza, other viral infections, and injuries	
Makowska et al. (2020) [43]	Self-Medication-Related Behaviors and Poland's COVID-19 Lockdown	- a purposely developed structured questionnaire<br>- online survey about participants' experiences with self-medication during three-month COVID-19 lockdown in Poland	- 45.6% of respondents indicated that during the lockdown they had engaged in inappropriate self-medication in at least one way<br>- 16.6% self-medicated as a precaution<br>- 16.8% self-medicated prescription medicine without consultation<br>- 19.4% of respondents bought a prescription medicine "just in case they might need it later"<br>- many respondents exhibited such behaviors for the first time during the lockdown and never engaged in this behavior prior to lockdown	Appropriate public health programs should be developed with the aim of helping people properly manage their medication in situations where the availability of physicians is limited such as threats of lockdown.
Aydın Aksoy et al. (2024) [47]	Nutrient Supplements for Young Children and Mothers' Self Medication with Over-the-Counter Drugs During the COVID-19 Pandemic	- descriptive study to evaluate mothers' use of nutrient supplements with over-the counter (OTC) drugs for their children (2- 6 years old) during COVID-19 pandemic	- 45.3% of mothers reported giving OTC medicines to their children<br>- mothers who frequently self-medicated themselves had 5.8 higher probability to self-medicate OTC drugs to their children – this association was significant	It is crucial that parents can acquire reliable health information and guidance on the safe use of OTC medications which is particularly important during health crises like the pandemic.

		- mothers were recruited through social media platforms	- mothers who self-medicated regularly before pandemic were more likely to give OTC medicines to their children than those who rarely or never self-medicated before the pandemic<br>- probability of administering OTC medicines to children was higher for children of 48-72 months (in comparison with children aged 24-47 months)<br>- most frequently used supplements were vitamin D, fish oil, multivitamins, vitamin C, immune boosters, zinc, probiotics, herbal teas, oral/nasal sprays, throat lozenges, and aspirin<br>- families with higher incomes had higher use of supplements<br>- delivery via Cesarean section had higher association with probability of supplements use<br>- reported primary purposes of self-medication were prevention and treatment	Administering OTC medicines to young children should be monitored with care to minimize risks. Continued education of the public is relevant to positively influence maternal decision-making concerning child health and medication practices.
King et al. (2022) [45]	Prevalence and correlates of stocking up on drugs during the COVID-19 pandemic: Data from the C3PNO Consortium	- two waves of interviews among participants who use drugs in nine prospective cohorts to estimate the prevalence and correlates of stockpiling drugs in the previous month during COVID-19 pandemic	- stockpiling of drugs was reported in 11.6% cases in the last month at baseline<br>- stockpiling of drugs was significantly and positively associated with being greatly impacted by COVID-19 and with at least daily use of methamphetamine in the past month	Addressing the impact of COVID-19 on vulnerable people who use drugs possibly could help limit drug stockpiling, which may decrease rates of high-intensity stimulant use.
Chaudhry et al. (2022) [19]	Factors Associated with Self-Medication during the COVID-19 Pandemic: A	- study performed through questionnaires, aimed to assess the characteristics, practices, and	- percentage of the participants who were tested for COVID-19 during the pandemic was 46.1%	It is important to establish awareness and understanding about the possible adverse effects of self-medication. In In

	Cross-Sectional Study in Pakistan	associated factors of self-medication by the public during the COVID-19 pandemic	- among them, 34.3% engaged in self-medication during the pandemic<br>- source of medicines in majority of cases was obtaining them directly from a pharmacy in 25.0% of cases<br>- health professionals had higher chances of practicing self-medication: 1.482 times greater for medical than for non-medical health personnel<br>- participants whose COVID-19 test was positive had probability of practicing self-medication 7.688 times higher than participants who did not test for COVID-19<br>- participants who had difficulty reaching physicians and difficulty in travelling/reaching healthcare professionals had 2.062-times and 1.862-times higher probability to practice self-medication respectively<br>- the most used medicines and supplements were acetaminophen (23.6%), azithromycin (14,9%), and cough syrups (13%), and OTC drugs, vitamin oral supplements, such as Vitamin C (39.1%), folic acid (23.5%), and calcium (22.6%)<br>- the usage of Vitamin C was significantly associated with being a healthcare professional or having a COVID test before<br>- observed self-medication practices were at an alarming level among study participants	addition, partnerships with pharmacists must be developed and applied in order not to sell prescription medicines without a verified prescription.

Mejia et al. (2023) [49]	Factors associated with the use of medicines and self-medication during the COVID-19 pandemic in 12 Latin American countries	- cross-sectional analytical study was conducted via an analysis of secondary data on the use of medicines and self-medication during COVID-19 pandemic collected from responses from 12 Latin American countries	- participants reported using paracetamol, ibuprofen, or antibiotics and disclosed this as self-medication in 26.9%, 16.6%, and 9.7% of cases respectively<br>- there were significant associations found between self-medication on individual's own choice reported and the use of hydroxychloroquine, antibiotics, or NSAIDs<br>- reported self-medication on the recommendation of a family member was associated with antibiotics, paracetamol, ibuprofen, or other NSAIDs<br>- self-medication on the recommendation of another person was associated with chloroquine, hydroxychloroquine, azithromycin, penicillin, other antibiotics, warfarin, ivermectin, paracetamol, ibuprofen, or other NSAIDs<br>- prevalence of self-medication in study population was significantly high, including the use of medicines not recommended for COVID-19 treatment and/or prevention	It is necessary to implement public health measures to fight against irresponsible self-medication to prevent negative impact on current health and future effectiveness of medicines particularly in case of antibiotics self-medication.
Arboleda Forero et al. (2023) [52]	Antibiotic Self-Medication Patterns and Associated Factors in the Context of COVID-19, Medellín, Colombia: A Survey Based Cross Sectional Study	- survey considering sociodemographic characteristics, self-medication with antibiotics, reasons for using these drugs, and types of antibiotics used in context of COVID-19	- self-medication with antibiotics was reported in 46% of cases<br>- 47.4% of this population reported using antibiotics without medical prescription usually for flu-like symptoms related to COVID-19<br>- the most used antibiotics were amoxicillin (33.7%), azithromycin (10.9%), and cephalexin (4.7%)	Increasing awareness of inappropriate self-medication with antibiotics and its possible consequences through targeted actions is important to reach the controlled use of these medications.

			- variables associated with self-medication were age group, zone of residence, and not enough information on the proper administration of medications used	
Zhang et al. (2021) [51]	Self-Medication with Antibiotics for Protection against COVID-19: The Role of Psychological Distress, Knowledge of, and Experiences with Antibiotics	- an online survey was conducted at the height of the initial outbreak and designed to address knowledge gap in using antibiotics in the context of the COVID-19 pandemic in Australia	- it was found that 19.5% of participants took antibiotics to protect themselves from COVID-19<br>- there was a positive association of pandemic and self-medication<br>- parameters associated with the use of antibiotics for prevention were lack of knowledge about antibiotics, practices of inappropriate antibiotic use, type of patient-physician relationship, demographic characteristics<br>- percentages of respondents who believed that antibiotics could treat common cold and viral infections were 18.9% and 28.1%, respectively<br>- 35.6% of respondents used antibiotics for cold/flu<br>- 23.2% of respondents used leftover antibiotics<br>- 47.1% of respondents reported it was easy to get antibiotics from physicians	There is a need to constructively manage self-medication with antibiotics and to focus on physician-patient interaction, prescribing practices and appropriate instruction on taking medicines to interrupt usual patterns of self-medication. Development and implementation of tailored education programs to raise awareness of risks of inappropriate self-medication is needed.
Mustafa et al. (2023) [54]	Knowledge, Attitude and Practices of Self-Medication Including Antibiotics among Health Care Professionals during the COVID-19 Pandemic in Pakistan: Findings and Implications	- descriptive cross-sectional study was conducted among health care professionals to assess their knowledge, attitude and practices of self-medication, including with antibiotics, during pandemic	-majority of participants had good knowledge and attitudes on self-medication, still 60% practiced self-medication during COVID-19 pandemic<br>- the most used medicines were antipyretics (100%)<br>- more than 1/3 of participants thought about self-medication as a part of self-care and they felt they could diagnose different diseases by themselves	Increasing the awareness of appropriate use of antibiotics to contain development of antimicrobial resistance development.

			- the most used drugs were antibiotics (80.4%) and vitamins (59.9%)<br>- the most purchased antibiotic was azithromycin in 35.1% of cases<br>- physicians and pharmacists had better knowledge of self-medication compared with nurses and other healthcare workers	
Salvador-Carrillo et al. (2024) [55]	Self-medication Practices During the Covid-19 Pandemic in a Latin American Country: A Cross-sectional Survey Study	- cross-sectional study was performed via e-survey that was distributed virtually to included population to describe the self-medication practices and associated risk factors among the Peruvian population during the COVID-19 outbreak	- reported frequency of self-medication among participants was 35.93%<br>- the most used drugs without a prescription in self-medicated population were ivermectin (drops, 72.01%), paracetamol (41.24%), and azithromycin (25.81%)<br>- factors associated with self-medication were living in Chincha*, divorced or widowed status, informal employment, having symptoms of COVID-19 with no diagnosis confirmed	There is a need to strengthen public health education, improve access to healthcare system and reinforce education of HCPs to diminish self-medication practices, especially during disastrous events such as pandemic.
Stüdemann et al. (2024) [46]	Individual use of self-medication and other remedies in COVID-19 outpatients in Western-Pomerania	- two questionnaires in an observational cohort study from positively tested COVID-19 outpatients to describe self-medication with OTC drugs and use of other remedies against symptoms of COVID-19	- the rate of participants who reported using medicines and other remedies to relieve COVID-19 symptoms was 74%<br>- most frequently used medicines were ibuprofen (26%), acetaminophen (21%), metamizole (14%), and acetylsalicylic acid (10%)<br>- participants reported frequent use of phytopharmaceuticals, herbal and animal products, and vitamins and minerals<br>- the most common symptoms for self-medication were headache	Further studies could be useful to examine a possible causal relationship between OTC medicines and COVID-19 disease course.

			(40%), other kinds of pain (e.g. myalgia; 37%), fever (24%) and cough (16%)	
Jifar et al. (2024) [50]	Assessment of factors associated with self-medication practices during the COVID-19 pandemic in southwestern Ethiopia: a community-based cross-sectional survey	- community-based cross-sectional study was performed at selected drug retail outlets for community pharmacy clients during the COVID-19 pandemic to assess factors associated with self-medication	- factors significantly associated with self-medication were female gender, having college education or above, waste of time in public health facilities, fear of getting COVID-19, high expenses in public health facilities<br>- the most frequently used medicines to treat or prevent COVID-19 were analgesics (42.4%), medicines for cold (29.5%), antibiotics (26.7%)<br>- the most frequently reported symptoms for self-medication with OTC medicines were headache (22.2%), cold (21.4%), fever (13.2%), allergy (5.2%)<br>- the most common symptoms for the use of antibiotics were respiratory infection (14.3%), cough (5.8%), sore throat (5.5%)<br>- more than one third of participants (36.6%) reported having medicines at home for treatment and prevention of COVID-19<br>- 26.4% of respondents reported using leftover medicines from previous treatments<br>- 86.4% of participants lack the knowledge about potential adverse reactions of medicines they used	Particular attention should be given to educate both HCPs and the public on the types of diseases which can be self-diagnosed and self-treated and the types of drugs to be used for self-medication.
Zheng et al. (2024) [57]	Patterns of self-medication and intention to seek pharmacist guidance among older adults during the COVID-19	- face-to-face cross-sectional survey was performed to assess the self-medication patterns and intention to seek pharmacist advice among older	- reported self-medication rate was 64.2%<br>- the most used medicines were over-the-counter and traditional medicines<br>- majority of respondents engaged in self-medication	To better control the risks associated with self-medication, pharmacists have the key role. Increasing the recognition and trust to pharmacists within society as well

	pandemic in Macao: a cross-sectional study	adults during COVID-19 pandemic	to prevent or treat COVID-19 symptoms<br>- the most common reason for self-medication was that disease was not perceived as serious enough<br>- variables significantly associated with self-medication were older age (> 85) and university degree<br>- older adults reported moderate intention to seek pharmacists' advice on the use of medicines	as adapting pharmacy governance models and building up pharmacists' self-perception of their profession are essential areas which should be addressed.
Souza et al. (2024) [56]	Prevalence and predictors of self-medication to prevent or treat COVID-19 among undergraduate students in Southern Brazil	- cross-sectional study was conducted among undergraduate students using an electronic questionnaire to evaluate prevalence and predictors of self-medication for the prevention or treatment of COVID-19	- reported prevalence of self-medication was 14.9%<br>- risk factors associated with self-medication were income between BRL 2101 and BRL 5250, studying at public university and studying a distance undergraduate course<br>- identified factors that protected against self-medication were age > 30 years, female gender, working or engaging in apprenticeships, sometimes recommending their own medicines to other people and deteriorating health during COVID-19 pandemic<br>- the main used products were ivermectin, vitamin c, vitamin D, tea, azithromycin, zinc and propolis	Health education measures should be developed and implemented to reduce self-medication among undergraduate students and guide the population regarding the risks of this practice.
Li et al. (2024) [53]	Prevalence of self-medication with antibiotics and its related factors among the general public and health professionals during the COVID-19 pandemic: A	- cross-sectional study was conducted to estimate the prevalence of self-medication with antibiotics and its associated factors in general public and health professionals during the COVID-19 pandemic	- the reported rate of self-medication with antibiotics was 10.25% in general population and 12.69% in HCPs<br>Findings for general population:<br>- variables associated with lower probability of self-medication in general population were perception of own health as average or	Since the results have shown that self-medication with antibiotics exists among the general population as well as HCPs, the efforts to increase awareness and promote the rational use of antibiotics require collective engagement.

	cross-sectional study in China		good, moderate antibiotic knowledge, and easy access to nearby health facilities<br>- variables associated with higher probability of self-medication were living in rural areas, found it easy to purchase antibiotics without prescriptions, and those who frequently encountered antibiotics recommended by pharmacy staff<br>For health care professionals:<br>- variables associated with lower probability to self-medicate were female gender, perception of own health as good, had moderate or high antibiotic knowledge, and had easy access to health facilities<br>- variable associated with higher probability of self-medication was easier way to purchase antibiotics without prescriptions	

* City severely impacted by COVID-19 with one of the highest mortality rates in Peru

Table S3. Overview of features of studies related to self-medication with antimicrobials (antibiotics)

Author(s) and Year	Study Title	Study Objectives and Design	Main outcomes	Implications
Väänänen et al. (2006) [62]	Self-medication with antibiotics--does it really happen in Europe?	- the study to determine whether antibiotics are used for self-medication in southern Spain y using a convenience sample	-use of antibiotics was reported by 28% of the respondents during the previous 6 months - 41% of antibiotic users bought antibiotics without a prescription<br>- the most common indication for antibiotic use was common cold in 45% of cases followed by sore throat in 17%	Importance of increasing AMR awareness and promoting rational and more supervised use of antibiotics to minimize the expansion of antibiotic resistance.
Grigoryan et al. (2006) [70]	Self-medication with antimicrobial drugs in Europe	- study surveyed the populations of 19 European countries to compare the prevalence of antimicrobial drug self-medication in	- the prevalence of actual self-medication varied from 1 to 210 per 1,000<br>- prevalence of intended self-medication varied from 73 to 449 per 1,000	Activities to diminish inappropriate self-medication should be directed towards prescribers, pharmacists and patients.

		previous year and intended self-medication and storage and to identify the associated demographic characteristics	- both rates were high in eastern and southern Europe and low in northern and western Europe<br>- as the most common reasons for self-medication respondents listed throat symptoms (e.g., dry, inflamed, red, or sore throat, inflamed tonsils, tonsil pain)<br>- as the main sources of medications respondents listed pharmacies and medication leftovers from previous prescriptions<br>- parameters associated with higher levels of self-medication were younger age, higher level of education, and presence of a chronic disease	
Roberts et al. (2020) [73]	Prevalence and dental professional awareness of antibiotic self-medication among older adults: Implications for dental education	- survey of prevalence and awareness of doctors of dental medicine of self-medication with non-prescribed antibiotics including general dentists, dental school faculty, and public health clinicians<br>- the second survey was distributed to households in an independent and assisted living facility	- the rate of respondents who reported the use of antibiotics in the previous 2 years was 68.3%<br>- the rate of respondents who reported self-medication with non-prescribed antibiotics was 6.4% for treating cold symptoms or pain<br>- the main reason for self-medication with antibiotics was the belief that antibiotics had previously resolved similar symptoms<br>- one-third of the older adult respondents didn't have the knowledge that antibiotics treat bacterial infections only<br>- approximately 1 in 16 older adults that were surveyed reported self-medication with antibiotics that were not prescribed<br>- dental professionals reported a certain level of knowledge of self-	There is the need for education on self-medication with antibiotics that were not prescribed to increase awareness, and it should be implemented in dental curricula.

			medication with non-prescribed antibiotics but were unaware of all origins	
Zhu et al. (2016) [67]	Self-medication practices with antibiotics among Chinese university students	- online survey was conducted using a pretested questionnaire to evaluate self-medication practices among Chinese university students	- 47.9% of participants reported a lifetime history of self-medication with antibiotics<br>- among students who reported self-medication with antibiotics 43.5% believed that antibiotics were appropriate for viral infections<br>- 65.9% of respondents had more than one episode of self-medication with antibiotics in the previous year<br>- 73.5% of respondents used at least two different antibiotics<br>- 57.1% of respondents changed antibiotic dosage during treatment<br>- 64.4% of respondents changed antibiotics during treatment<br>- independent risks to engage in self-medication were female gender, older age, and prior knowledge of antibiotics<br>- 13.3% of respondents who reported self-medication with antibiotics had adverse drug reaction<br>- as independent risk practices associated with an adverse drug reaction identified were frequent change of dosage and concomitant use of the same antibiotic with different names	It is necessary to have firm regulations on antibiotic sales. In addition, recommended is public education supported by further health care reform.
Wang et al. (2018) [63]	Keeping Antibiotics at Home Promotes Self-Medication with Antibiotics among Chinese	- survey with self-administered online questionnaire among university students who were selected using cluster random	- the rate of surveyed students who reported keeping antibiotics at home at the time of survey was 63.1%	Findings about the practices of keeping antibiotics at home present a serious issue which suggest there is a need for professional

	University Students	sampling from the selected universities to evaluate the role of keeping antibiotics at home as driver of self-medication with antibiotics	- 27.8% of those who kept antibiotics in their home, reported that these antibiotics were left over from a previous prescription by a doctor and 69.3% purchased antibiotics over the counter<br>- 29.6% of students self-medicated with antibiotics when sick during the month before the survey<br>- for students who reported keeping antibiotics at home it was five times more probable to engage in self-medicating antibiotics when sick and 2.6 times more probable to self-medicate with antibiotics for prophylaxis than the other students - the variables associated with significantly higher probability of keeping antibiotics at home were female gender, those with a family background of higher education, and those who had a parent working in the medical field	directives and population-tailored health education.
Bi et al. (2000) [64]	Family self-medication and antibiotics abuse for children and juveniles in a Chinese city	- study to assess self-medication and misuse of antibiotics in families with questionnaire for parents of children in kindergarten, primary school and secondary school	- the reported rate of parents who self-medicated their children was 59.4%<br>- rate of self-medication increased with children's age<br>- approximately 51% of children had received self-medication from their parents on six or more occasions during the previous 1year and 32.8% on four to five occasions<br>- the association was found between parents who self-medicate and sources of medicine and severity of disease<br>- rate of inappropriate antibiotics use was 35.7%	More education on proper antibiotic use and on possible risks associated with irresponsible self-medication of antibiotics are needed.

			- there were significant associations found between self-medication and payment of the mother's medical fees by employers, severity of diseases as well as the mother's educational level	
Napolitano et al. (2013) [72]	Public knowledge, attitudes, and experience regarding the use of antibiotics in Italy	- cross-sectional survey with self-administered questionnaire was conducted on a random sample of parents of students attending randomly selected public primary and secondary schools to evaluate public knowledge, attitudes and experience considering the use of antibiotics	- the rate of parents who were aware of the definition of antibiotic resistance was 9.8%<br>- the rate of parents who were aware of when it was appropriate to use antibiotics was 21.2%<br>- higher probability of knowing the definition of antibiotic resistance was found for respondents with higher education, employed, with a family member working in the health care sector, and with no need for additional information on antibiotics<br>- the rate of participants who listed themselves as users of self-medication was 32.7%<br>- participants with higher probability of using self-medication were those with a lower self-rated health status, who did not use the physician as source of information on antibiotics, and who have attended a physician in the last year<br>- 22.7% of participants who had never been self-medicated would be inclined to use an antibiotic without a prescription of a physician<br>- factors associated with higher inclination towards the use of antibiotics without prescription from physicians were age < 40,	Functional public health initiative should be implemented to ensure feasible and suitable mechanisms to change the practices of irresponsible self-medication.

			lower self-rated health status, lack of knowledge that antibiotics are not indicated for treating flu and sore throat, and having knowledge that antibiotics are not indicated for treating colds	
Pedrolongo et al. (2024) [61]	Study on the Consumption of Non-Steroidal Anti-Inflammatory Drugs and Antibiotics by the Brazilian Adult Population: A Cohort Study	- the study was conducted via online questionnaire on the consumption of antibiotics and NSAIDs by the adult population considering the 90 days prior to the survey	- the rate of respondents who used NSAIDs was 89.5% and for antibiotics it was 32.2% whether or not these medications had been prescribed by doctors or dentists<br>- the prevalence of NSAIDs consumption in the studied population was high which is consistent with the high frequency of risk of adverse reactions caused by these drugs, especially in the gastrointestinal tract<br>- it was observed that the non-prescription use of antibiotics by the population was considered high, reaching one-third of the total number of volunteers who used these medicines	Furthermore, strengthen the education of HCPs who prescribe and dispense antibiotics to contain self-medication to prevent even bigger scale of the problem.
Pei et al. (2023) [68]	The Role of Uncertainty and Negative Emotion in Chinese Parents' Self-Medication of Children with Antibiotics	- a study to evaluate the influence of uncertainty and negative emotions in parents' self-medication of children with antibiotics<br>- responses were collected through an online survey from parents of children 6 - 12 years old	- the rate of respondents who reported they were engaged in self-medication with antibiotics to their children was 66.5%<br>- the most frequently used antibiotics for children were amoxicillin, cephradine, and azithromycin<br>- uncertainty was positively associated with negative emotions, which were in turn positively associated with attitude toward self-medication with antibiotics<br>- uncertainty was also negatively associated with perceived behavioral control (PBC), but the	Understanding the psychological factors driving parental self-medication with antibiotics may inform customized interventions to promote responsible antibiotic use among parents.

			association between PBC and self-medication with antibiotics behavior was not significant<br>- attitude and subjective norm were both positively associated with self-medication with antibiotics	
Cruz et al. (2022) [74]	Factors associated with self-medication of antibiotics by caregivers in pediatric patients attending the emergency department: a	- case-control study of factors connected to self-medication of antibiotics based on surveys of caregivers of pediatric patients brought to the emergency room with clinical symptoms suggestive of acute infection<br>- information was collected through a self-administered questionnaire	- higher parental education was related to less self-medication<br>- higher probability of self-medication was related to attitudes such as always requesting antibiotics from their physicians, frequently purchasing antibiotics without a prescription, and giving advice on antibiotics among family members<br>- higher probability of self-medication with antibiotics was observed in older children, children with greater number of siblings and in children who received antibiotics 3 months prior to survey<br>- knowledge about the risks of self-medication with antibiotics was low	These findings will inform future interventions to reduce self-medication in children.
Saif et al. (2024) [65]	Cross-sectional survey to explore knowledge, attitude, practices and impact of an intervention programme related to antibiotic misuse and self-medication among general population of Pakistan	- a cross-sectional quantitative study on knowledge, practices, attitude and adherence to antibiotic therapy was conducted among adult population<br>- individuals with poor knowledge were selected for video-based intervention programs<br>- a post-intervention study was conducted to assess the improvement	- 39.2% of participants had self-medicated antibiotics in the previous 6 months<br>- 42% of participants were non-adherent to antibiotic treatment plan<br>- the most important barrier in adherence to antibiotic treatment was lack of proper information from HCPs<br>- the informative video was shown to 31.64% of participants who didn't have adequate knowledge of the use of antibiotics and possible risks connected with it	Specific relevant measures are alarmingly needed to prevent antibiotics to become futile in eliminating various controllable microbial diseases.

			- the postintervention part of the study showed there was significant improvement in mean scores of knowledge, practices, attitude and adherence related to antibiotics	
Jifar et al. (2024) [50]	Assessment of factors associated with self-medication practices during the COVID-19 pandemic in southwestern Ethiopia: a community-based cross-sectional survey	- community-based cross-sectional study was performed at selected drug retail outlets for community pharmacy clients during the COVID-19 pandemic to assess factors associated with self-medication	- factors significantly associated with self-medication were female gender, having college education or above, waste of time in public health facilities, fear of getting COVID-19, high expenses in public health facilities<br>- the most frequently used medicines to treat or prevent COVID-19 were analgesics (42.4%), medicines for cold (29.5%), antibiotics (26.7%)<br>- the most frequently reported symptoms for self-medication with OTC medicines were headache (22.2%), cold (21.4%), fever (13.2%), allergy (5.2%)<br>- the most common symptoms for the use of antibiotics were respiratory infection (14.3%), cough (5.8%), sore throat (5.5%)<br>- more than one third of participants (36.6%) reported having medicines at home for treatment and prevention of COVID-19<br>- 26.4% of respondents reported using leftover medicines from previous treatments<br>- 86.4% of participants lack the knowledge about potential adverse reactions of medicines they used	Particular attention should be given to educate both HCPs and the public on the types of diseases which can be self-diagnosed and self-treated and the types of drugs to be used for self-medication.

Nazari et al. (2024) [66]	Prevalence and determinants of self-medication consumption of antibiotics in adults in Iran: a population based cross-sectional study, 2019-2020	- population-based cross-sectional study was conducted and analyzed self-reported annual consumption of antibiotics, as well as a record of antibiotic use registered in insurance services during the same period to assess prevalence and key factors of self-medication with antibiotics of adults	- reported annual prevalence of antibiotic self-medication was 30.3%<br>- significant correlation was found between educational level and self-medication practices<br>- no significant relations were found between self-medication practices services and gender, occupation, insurance coverage, or marital status<br>- there was a lower probability for female respondents to engage in self-medication<br>- there was a reduced probability found for self-medication of antibiotics and older age and higher educational level	It is necessary to increase the awareness of self-medication with antibiotics and AMR through development and implementation of inclusive public health strategies and coordinated efforts of HCPs.
Duan et al. (2024) [75]	Eliciting parents' decision-making to antibiotic use for upper respiratory tract infections: A discrete choice experiment	- a systematic review was conducted and in-depth interviews to identify the key attributes of choices when parents self-medicate antibiotics for children's upper respiratory tract infections	- symptom severity was the most important in parents' decision-making to self-medicate antibiotics followed by the risk of side effects or resistance, duration, total cost, onset time of antibiotic, and antibiotic effectiveness<br>- parameters significantly associated with higher probability for parents to self-medicate their children with antibiotics for URTIs were more severe and longer symptoms, perceived higher effectiveness, and fewer side effects of antibiotics	There is a need to develop sophisticated intervention strategy to reinforce parents' ability to differentiate mild from severe upper respiratory tract infections as well as their knowledge of antibiotics.
Darakhvelidze et al. (2024) [76]	Self-medication with antibiotics in Georgian population	- a cross-sectional study was performed using a random sampling method and developed self-administered questionnaire to collect data on self-	- total prevalence of self-medication was 32.6%<br>- the rate of self-medication of adults with antibiotics was 23.8%<br>-12.7% confirmed the self-medication of antibiotics	The level of knowledge of inappropriate self-medication with antibiotics should be increased through public awareness campaigns and implementing

		medication with antibiotics<br>- the survey was conducted online	without medical advice to treat minor family members	functional interventions to contain the sales of antibiotics without physician's prescription.
Elhaddadi et al. (2024) [69]	Prevalence and Determinants of the Use of Antibiotics by Self-Medication in the Pediatric Population in Oujda, Morocco	- a cross-sectional study conducted using a questionnaire-guided interview with parents of children consulting or hospitalized in the Pediatric Department to assess determinants of self-medication with antibiotics in pediatric population	- self-medication with antibiotics was reported in 68% of included families<br>- main symptoms for self-medication with antibiotics were cough (43%) and fever (24%)<br>- most self-medicated drugs by parents were betalactams (72%)<br>- majority of information on proper use of antibiotics (dosage and methods) was provided by pharmacy staff but was not respected by most parents (78%)<br>- the main reasons reported for using antibiotics by self-medication were the efficacy of treatment (58%), saving consultation costs and time (47%), and the ease of obtaining treatment without a prescription (42)<br>- the probability of self-medicating with antibiotics in the pediatric population was increased by female gender, low level of education, low socio-economic status and buying antibiotics without a prescription	It is necessary to promote a global health strategy.

Table S4. Overview of features of studies related to self-medication in health care professionals or students of healthcare studies

Author(s) and Year	Study Title	Study Objectives and Design	Main outcomes	Implications
Aşut et al. (2025) [79]	Is self-medication a barrier to seeking help: exploring psychiatrists' and psychiatry	-descriptive cross-sectional study via anonymous online questionnaire to estimate psychiatrists' and psychiatry	- the reported rate of self-medication in the previous year was 83%<br>- the most common self-medicated medicines were antidepressants	There is a need for educational improvements and better support systems that could enhance help-seeking which

	residents' knowledge and attitudes about self-medication in Türkiye	residents' knowledge and attitudes about self-medication in Türkiye	- major reported reason for self-medication was viewing problems as minor, previous positive experiences with self-medication and time constraints for seeking help<br>- many respondents were unaware of legal and ethical guidelines	finally should result in better health outcomes for psychiatrist and psychiatry residents.
Balon (2007) [81]	Psychiatrist attitudes toward self-treatment of their own depression	- survey based on mailed questionnaire asking whether the psychiatrist would or did self-treat for depression	- the rate of respondents who would consider self-medication or self-medicate if afflicted with mild/moderate depression was 43%<br>- the rate of respondents who would self-medicate or consider self-medication for severe depression or if suicidal ideation became a component of one's depression was 7%<br>- the rate of respondents who self-medicated themselves for depression in the past was 15.7%	A substantial number of psychiatrists would treat themselves for depression, perhaps because of fear of stigma or a permanent record, or other reasons.
Kim et al. (2025) [82]	Self-prescription of opioids, sedative-hypnotics, and other psychotropic medications among physicians in South Korea	- data on self-prescription of physicians with opioids, sedative-hypnotics, and other psychotropic medications in South Korea from 2020 to 2023 were obtained from the Ministry of Food and Drug Safety	- about 7% of practicing physicians self-prescribed opioids, sedative-hypnotics, or other potentially habit-forming drugs<br>- it was less than 1/5 of prescription of these types of drugs among the general population<br>- the most commonly self-medicated drug was zolpidem<br>- frequently prescribed were also other sedatives, anxiolytics, and appetite suppressants<br>- the prevalence of self-prescription among physicians was lower than the prevalence of similar prescriptions in the general population	Since self-prescribing for some physicians could relate to use of more possibly habit-forming drugs than necessary, closer surveillance of such self-prescribing habits among physicians may be needed.

			- on the other hand, the number of pills of these medications per physician who self-prescribed was higher than that prescribed in the general population	
Rosvold et al. (2002) [97]	Illness behaviour among Norwegian physicians	- mailed, anonymous questionnaire as a part of the Norwegian Medical Association's health survey to evaluate behavior among physicians	- the percentage of respondents who performed self-treatment during the last three years was 75%<br>- treatment by spouse, friends, and colleagues was also frequent<br>-among participants who used prescription medicine, 73% were self-prescribing it<br>- 13% of the physicians had negative experiences with self-treatment<br>- approximately 56% reported that they would prefer to seek help for somatic problems from a physician to whom they had no personal ties<br>- 50% stated they would seek help with mental problems elsewhere than in the mental health care facilities located where they lived	Since many physicians practice self-medication when they are ill and may contact friends and colleagues when they need help from another physician it is worth addressing situations like these during their education.
Benameur et al. (2019) [89]	Knowledge, attitude, behaviour of the future healthcare professionals towards the self-medication practice with antibiotics	- cross-sectional study based on survey among students using validated questionnaires to evaluate knowledge, attitude, behavior of the future healthcare professionals towards the self-medication practice with antibiotics	- reported prevalence of self-medication with antibiotics was 58.4%<br>- proportion of self-medication with antibiotics was significantly lower among medical students<br>- the most common symptom for self-medication with antibiotics was tonsillitis and it was reported by a significantly higher proportion of medical students (54.1%)<br>- the rate of awareness of medical students that self-medication with antibiotics	Health education should be improved in the direction of addressing this issue in a way which would result in ameliorating students' knowledge, awareness and attitudes on the use of antibiotics.

			is not safe and is inappropriate was 79.9%<br>- the prevalence of self-medication with antibiotics remains high<br>- higher probability of using antibiotics by self-medication was found in students who incorrectly, identified the effectiveness of antibiotics in treating bacterial infections and the reasons of the antibiotics' discontinuation	
Turan (2024) [90]	Self-Medication of Medical Students at Erzincan Binali Yıldırım University in Turkey: A Cross-Sectional Study	- cross-sectional study with questionnaire included first-, fourth-, and sixth-year medical students to evaluate practices of self-medication of medical students in Turkey	- the rate of self-medication among participants was 96.1%<br>- there was no statistically significant difference found in rates of self-medication based on the year of study<br>- the reported rate of students using antibiotics without a prescription was 19.3%<br>- the percentage of students reported reading the package insert before using a drug was 82.5%<br>- the most common symptoms for engaging in self-medication were headaches (83.1%) and the common cold (63.6%)<br>- most frequently self-medicated drugs were analgesics (86.1%), medicines for common cold (54.8%), and vitamins (34.0%)<br>- reported reasons for self-medication were perceiving their condition as a simple illness (79.3%), experiencing a similar illness before (64.6%), and belief they had sufficient knowledge of medications (28.2%)	Educational interventions to promote the development of responsible self-medication behaviors have an important role and should be a part medical school education from the first year to significantly enhance relevance of responsible practices of self-medication.

			- keeping medicines at home/in the dormitory for later use and not having chronic disease were found to be effective factors in engaging in self-medication practices	
Galán Andrés et al. (2021) [88]	Self-Medication of Drugs in Nursing Students from Castile and Leon (Spain)	- descriptive study was performed among nursing students in Spain to get insight into self-medication among them	- the rate of respondents who reported using non-prescribed medicines in the previous month was 73.8%<br>- the reported reason for self-medication was familiarity with the health problem and its pharmacological solution in 28.9% of respondents and belief that the health problem was mild in 25% of respondents<br>- most frequently used drugs off-prescription were analgesics in 88.91% of occasions<br>- drugs were mainly recommended by the students' family in 58.12% of the cases<br>- students reported keeping analgesics they take without prescription in their home first aid kit<br>- 53.2% of respondents obtained drugs without prescription from the home first aid kit<br>- respondents reported giving advice and recommending drugs they have taken to other people with similar symptoms<br>- 85.72% students reported keeping excess drugs after a treatment<br>- self-medication was related to keeping unused medicines and providing advice on the use of drugs to other people	There is a need to review and improve education on rational medicines use and responsible self-medication in the curriculum.

Al Essa et al. (2019) [91]	Practices, awareness and attitudes toward self-medication of analgesics among health sciences students in Riyadh, Saudi Arabia	- cross-sectional study in a form of electronic questionnaire survey was conducted among health sciences students in Saudi Arabia to evaluate their practices, awareness and attitudes toward self-medication of analgesics	- high prevalence of self-medication was reported (73.2%)<br>- approximately 70% of respondents reported that their drugs were not prescribed by physician<br>- 21% reported that self-medication was their first method of treatment for all problems<br>- 34% of respondents indicated they have adequate knowledge on drugs and diseases<br>- as reasons for self-medication respondents choose willingness to play an active role in their health (47.4%), not inclined to visit physicians due to a long waiting time (39%) and having adequate knowledge of medication and disease (34.2%)<br>- symptoms for which self-medication was practiced were minor illness such as headache (92%), fever (52.2%) or menstrual cramps (43.8%)<br>- approximately 92% of respondents were aware of the adverse effects connected with analgesics use<br>- most frequently used analgesics were paracetamol (96.5%) and NSAIDs (49.1%)	It is necessary to educate students about responsible self-medication as well as about the risks of irresponsible self-medication.
Alduraibi et al. (2022) [92]	A cross-sectional survey: knowledge, attitudes, and practices of self-medication in medical and pharmacy students	- cross-sectional study was conducted to estimate knowledge, attitudes, and practices of self-medication in medical and pharmacy students<br>- data was collected through questionnaire	- the majority (94.6%) of students had good knowledge of self-medication<br>- good knowledge, was significantly associated with being female, and pharmacy students	The importance of medical and pharmacy students as contributors to the public health care system, and future health professionals should be reflected in proper education on responsible self-

			- 63.9% of the students reported practicing self-medication in the last 6 months<br>- the most common medications used for self-medication by most of the students were analgesics in 88.29% of cases	medication and good pharmacy practice.
Kokabisaghi et al. (2024) [96]	The prevalence and causes of self-medication among medical university students in Iran during COVID-19 outbreak and its implications for public health and health systems: A cross-sectional study	- descriptive-analytical cross-sectional study among the students of health sciences programs which evaluated the knowledge, attitude, and practice of self-medication during COVID-19<br>- data collection tool included a questionnaire designed online and available to students	- the reported rate of self-medication among medical university students was 19%<br>- as the most common reason for self-medication respondents identified the safety of medicines (66.67%)<br>- the most usual form of medication used was the tablet (35.67%)<br>- the most common medication taken was paracetamol (69.01%)<br>- the most common symptom to engage in self-medication was headache (67.36%)<br>- variables associated with self-medication were age and type of degree<br>- older participants and postgraduate students had positive attitudes toward self-medication	Recognizing the importance of the role that medical students will have in the future in the field of medication prescribing and self-medication, it is necessary to pay more attention to their education in this area together with controlling the prevalence of self-medication.
Hassan et al. (2025) [93]	Self-medication pattern among medical students in Middle Delta, Egypt	- cross-sectional study was carried out among undergraduate medical students from the first to final year to assess self-medication patterns<br>- data was collected using a predesigned semi-structured self-administered questionnaire	- the prevalence of self-medication was 71%<br>- causal factors associated with self-medication were students' medical knowledge from self-experience and studies (55.9%)<br>- common symptom for self-medication was headache (80.4%)<br>- majority of respondents (88.3%) use pharmaceutical products	It is necessary to value diagnosis, awareness and seriousness of self-medication.

			- most common medicines respondents self-medicated were analgesics (92.4%)<br>- the primary reason for drug selection was the degree of popularity of medicine (52.5%)<br>- the main determinate of selecting type of drug was the recommendation of the pharmacist (43.6%)<br>- rate of respondents who experienced adverse drug reactions was 30.2% of whom 50% went to private physicians and 33.5% stopped taking their medications<br>- half of the students took antibiotics for self-medication	
Jayawardhana et al. (2023) [94]	Investigating the drivers for antibiotic use and misuse amongst medical undergraduates-perspectives from a Sri Lankan medical school	- cross-sectional study was conducted among medical students to explore key factors that promote antibiotic use and misuse amongst medical students<br>- a validated, self-administered Google forms-based online questionnaire was used	- 46.4% of participants reported the use of antibiotics without a prescription<br>- 32.0% reported keeping left-over antibiotics for future use; 23.3% - not completing the course of antibiotics, 21.3% - use of left-over antibiotics, 17.6% - prescribing to animals; 14.7% - prescribing antibiotics to family members or friends; 7.2% antibiotic self-medication; 6.9% - not following the dosage regime prescribed<br>- antibiotic prescription by medical undergraduates was perceived as unacceptable by the majority (94.8%)<br>- individual misuse patterns were associated favorably or unfavorably with gender, year of study, having a health care worker -the knowledge score increased	Since the self-medication practices remain the same over the years, although students advanced considering knowledge, the need was revealed to identify the additional drivers of antibiotic misuse among medical undergraduates.

			with the advancement in training at the medical school while the practice score remained indifferent	

Table S5. Overview of features of studies related to self-medication and storing medicines at home

Author(s) and Year	Study Title	Study Objectives and Design	Main outcomes	Implications
Louhisalmi et al. (2025) [105]	Amount, type and storage of medicines in households - A survey for medicine users	- the online survey was conducted among loyal customers of University Pharmacy to determine the amount, type and storage practices of drugs in households<br>- also investigated were the reasons for unnecessary or expired medicines, as well as the determinants associated with the presence of expired medicines in a household	- typically, drugs were stored in the kitchen (67.0%) and in cabinets (58.7%)<br>-40% of drugs were to be stored safely<br>-the main reasons for keeping unnecessary or expired medicines were improved health (39.2%), medication changes (31.9%) and oversized packs (28.0%)<br>- expired medicines were more frequently found in households with many active medicines	The study showed that households had many medicines, highlighting the need for better storage and optimized packaging to improve safety, reduce waste and enhance rational pharmacotherapy.
Tieu Mai Diep et al. (2024) [102]	Prevalence and determinants of household medicine storage in Vietnam: A community-based cross-sectional study. SAGE	- a community-based cross-sectional study was conducted with 800 households to research the prevalence of and identify the factors that influence medicine storage in Vietnamese households and medicine storage practice	- drugs were found in 71.6% of surveyed household<br>- the most common type of medicine kept were analgesics-antipyretics (80.8%)<br>- the main source of getting medicines was private pharmacies in 90.1% of households<br>- 68.1% of households stored medicine for future use<br>- 58.8% of households had a home medicine cabinet<br>- 9.4% of households did not keep medicine in the appropriate packaging<br>- 19.4% of households did not check the expiry date of their medicine<br>- key determinants of keeping the medicines were educational level, income, presence of chronic	Considering a high prevalence of household medication storage and some inappropriate storage behaviors, attention should be given to the development of effective interventions and policies to promote safe and appropriate storage practices.

			illnesses, presence of children, presence of healthcare professionals	
Volkos et al. (2024) [107]	Psychotropic medicine use without current or prior medical advice, storage at home and association with perceived stress in a primary health care setting in Greece	- study to assess the prevalence of self-medication of psychotropic medicines and supplements without medical advice, including storage at home,<br>- cross-sectional sample of adult attendees were surveyed during regularly scheduled appointments during a three-week period	- 38.4% of respondents reported having psychotropic medicines stored at home medical cabinets<br>- 27.4% practiced self-medication with psychotropic medicines in the last 12 months	The findings could potentially inform primary care providers to focus on patients most likely to be users of PMS without medical advice.
Köksoy (2024) [103]	Unused, expired pharmaceuticals and their disposal practices among the general public in Burdur-Türkiye: a cross-sectional study	- a cross-sectional study to identify unused pharmaceuticals, determine the number of unused medicines, analyze practices of disposal methods, classify the medicines according to Anatomical Therapeutic Chemical classification (ATC)	Findings of first sample group: -drugs were stored in kitchens (59.1%) and refrigerators (38.6%)<br>-the reason for keeping drugs was reuse (41%)<br>-drugs were disposed in household garbage (81%)<br>-the most frequently unused pharmaceuticals were paracetamol, preparations for cold, dexketorpofen, diclofenac, amoxicillin and betalactamase inhibitor<br>- self-medication of unused medicines at home was reported in 94.1% of cases<br>Findings of second sample group: -50.6% of found drugs were used, and 49.4% were found to be unused	Since there was a significant amount and number of unused medicines as well as expired and not properly stored medicines found in households, together with common practice of self-medication there is an obvious need for increasing public awareness on inappropriateness of these practices and giving directions towards responsible medicines use and disposing.
Beuscart et al. (2019) [106]	Polypharmacy in older patients: identifying the need for support by a community pharmacist	- a study conducted through interviews with older adults (aged 65 or over) taking at least five medications daily<br>-the student interviewer also offered to examine the patient's home medicine cabinet	- in 54.2% of interviews conducted examinations of the home medicine cabinet were also included<br>-adverse events were reported 42.0% of participants<br>-27.6% of participants reported difficulties in preparing, administering and/or swallowing medications	Pharmacists working in community pharmacies could identify high-priority older patients that need help with managing medications by asking simple questions about difficulties in managing, administering, taking or storing medications.

			-home medicine cabinets were poorly located in 15.0% of inspections -medication storage problems were reported in 21.7%<br>-expired medications were found in 40.7% of cases<br>-potentially inappropriate medications were found in 15.0%<br>-several different generic versions of the same drug were found in 19.9%<br>-redundant medications were found in 20.4%	
De Bolle et al. (2008) [101]	Home medication cabinets and self-medication: a source of potential health threats?	-a cross-sectional study was conducted in Belgian community pharmacies<br>-adult pharmacy customers (aged 18-80 years) were visited in their homes by pharmacy students<br>- hose medical cabinets were inspected, and the participants were interviewed	- in 1/3 of the households drug packages were not stored safely<br>- the percentage of prescription drugs was 34%<br>-the most frequently found medicines were nonopioid analgesics (7.2%), NSAIDs (6.9%), nasal decongestants (3.5%) and antinausea agents (3.2%)<br>- despite their high prevalence NSAIDs and non-opioid analgesics were not predominant among the most frequently used drugs<br>- in 23.3% of households food supplements were used daily<br>-percentage of found expired drugs was 9%<br>-percentage of drugs that were not stored in the original container, and the package insert was missing was 18%<br>- the rate of self-medication was 56% for all drugs (OTC drugs - 74%; prescription drugs - 21%)<br>- when consulting the package insert only 5.2% of the participants misjudged indication, dosage, or treatment duration	Since many younger participants showed inclination towards self-medication, there is a justified need to develop awareness of the risks of self-medication to avoid the risks connected with it.

			-the willingness to self-medication decreased with age and with increasing number of drugs taken daily	
Tourinho et al. (2008) [104]	Home medicine chests and their relationship with self-medication in children and adolescents	- a descriptive population study based on a home survey of a simple random sample<br>-participants ≤ 18 years, an obligatory interview with at least one guardian, inventory of medicines kept at home; and having taken at least one drug in prior 15 days before the interview<br>-participants were divided into 2 groups based on medication use – medical prescription and self-medication (lay advice)	-79.6% of total medicines found in households were pharmaceutical preparations<br>-the most frequently drugs were stored in bedrooms (47.5%), kitchens (29.9%), and bathrooms (14.6%)<br>-76.5% of medicines were in cardboard boxes and within easy reach of children aged ≤ 6 years<br>-the most frequently used drugs were analgesics/antipyretics (26.8%) and systemic antibiotics (15.3%)<br>-significantly larger hoards of these medications were found in self-medication group<br>-factors connected to self-medication group with higher probability of self-medication were storing medications in the bathroom and legal guardians with ≤ 4 years of primary education	To encourage rational use, minimize waste and stimulate safe storage of drugs it is necessary to develop and implement awareness campaigns.
Aljinović-Vučić et al. (2005) [95]	Content of home pharmacies and self-medication practices in households of pharmacy and medical students in Zagreb, Croatia: findings in 2001 with a reference to 1977	-cross-sectional anonymous questionnaire-based survey to assess the content of household drug supplies and self-medication practice<br>- healthcare students (medicine and pharmacy) inventoried medical cabinets in their family households and interviewed the household members on drug keeping and self-medication practice	- in every household at least one drug was found<br>-in 68% of the households drugs were kept at a designated place<br>--drugs past expiry dates and/or with purpose unknown to the household members were reported in 27% of the households<br>-drugs most frequently found in households were NSAIDs (97%) followed by antibiotics (46%)<br>-in households with NSAIDs they were self-medicated in 88% of cases and self-medication of antibiotics was practiced in	There is a need to increase awareness on responsible self-medication and enhance education in irresponsible self-medication of antibiotics.

		37% of the households in which they were found	
